# Predictive P-score for treatment ranking in Bayesian network meta-analysis

**DOI:** 10.1186/s12874-021-01397-5

**Published:** 2021-10-17

**Authors:** Kristine J. Rosenberger, Rui Duan, Yong Chen, Lifeng Lin

**Affiliations:** 1 Department of Statistics, Florida State University, 411 OSB, 117 N Woodward Ave, Tallahassee, FL 32306 USA; 2 Department of Biostatistics, Harvard University, Boston, MA USA; 3 Department of Biostatistics, Epidemiology and Informatics, University of Pennsylvania, Philadelphia, PA USA

**Keywords:** Bayesian analysis, Heterogeneity, Network meta-analysis, P-score, Prediction, Treatment ranking

## Abstract

**Background:**

Network meta-analysis (NMA) is a widely used tool to compare multiple treatments by synthesizing different sources of evidence. Measures such as the surface under the cumulative ranking curve (SUCRA) and the P-score are increasingly used to quantify treatment ranking. They provide summary scores of treatments among the existing studies in an NMA. Clinicians are frequently interested in applying such evidence from the NMA to decision-making in the future. This prediction process needs to account for the heterogeneity between the existing studies in the NMA and a future study.

**Methods:**

This article introduces the predictive P-score for informing treatment ranking in a future study via Bayesian models. Two NMAs were used to illustrate the proposed measure; the first assessed 4 treatment strategies for smoking cessation, and the second assessed treatments for all-grade treatment-related adverse events. For all treatments in both NMAs, we obtained their conventional frequentist P-scores, Bayesian P-scores, and predictive P-scores.

**Results:**

In the two examples, the Bayesian P-scores were nearly identical to the corresponding frequentist P-scores for most treatments, while noticeable differences existed for some treatments, likely owing to the different assumptions made by the frequentist and Bayesian NMA models. Compared with the P-scores, the predictive P-scores generally had a trend to converge toward a common value of 0.5 due to the heterogeneity. The predictive P-scores’ numerical estimates and the associated plots of posterior distributions provided an intuitive way for clinicians to appraise treatments for new patients in a future study.

**Conclusions:**

The proposed approach adapts the existing frequentist P-score to the Bayesian framework. The predictive P-score can help inform medical decision-making in future studies.

**Supplementary Information:**

The online version contains supplementary material available at 10.1186/s12874-021-01397-5.

## Background

Network meta-analysis (NMA) of randomized controlled trials is a statistical method widely used to draw conclusions about multiple treatment comparisons in evidence-based medicine [1–7]. It simultaneously synthesizes both direct and indirect evidence, where the direct evidence comes from head-to-head trials and the indirect evidence comes from indirect comparisons with common treatment comparators. For example, the comparison between two active drugs A and B can be informed from the indirect comparisons of A vs. C and B vs. C, where C may be a placebo, or from the direct comparison in clinical trials comparing A vs. B. The synthesis of both direct and indirect evidence can provide more precise estimates of treatment effects (i.e., estimates with smaller variances and thus narrower confidence or credible intervals) than conventional meta-analyses that compare pairs of treatments separately [8–11].

One of the main purposes of NMAs is finding a succinct way to present summarized results among many competing treatment options and inform future clinical trial designs. Rank probability (i.e., the probability of a treatment having a certain rank *r*) and cumulative rank probability (i.e., the probability of a treatment being at least the *r* th best) are commonly used measures for treatment rankings in an NMA. A limitation to these ranking methods is that they do not yield a simple single number to summarize the rank of each treatment [12]. When many treatments are available for a certain disease, these may be hard to interpret and may not be useful measures to clinicians [13]. Some researchers might rely only on the probability of being the best treatment for decision-making, which could draw misleading conclusions. It may be more intuitive to summarize the multiple probabilities into a single number for each treatment to assess the overall performance of the treatments.

One such summarization method is the surface under the cumulative ranking curve (SUCRA) proposed by Salanti et al. [12]. The SUCRA is calculated by averaging cumulative rank probabilities; it essentially transforms the mean rank of a treatment to a value between 0 and 1 [14]. The SUCRA is advantageous over the mean rank because its common range from 0 to 1 facilitates consistent interpretations across different NMAs, while the mean rank depends on the number of treatments in a network. The higher value of the SUCRA indicates a better treatment; SUCRA = 0 or 1 indicates an always worst or best treatment, respectively.

A relevant concept is the P-score, which was originally proposed under the frequentist framework by Rücker and Schwarzer [14]. The P-score of a treatment is obtained by estimating the effect sizes of pairwise treatment comparisons and assuming their point estimates are normally distributed. Like the SUCRA, the P-score ranges from 0 to 1, with 0 or 1 being the theoretically worst or best treatment, respectively. Although it is defined differently from the SUCRA, the P-score has been shown to be identical to the SUCRA under the frequentist framework [14].

These approaches to treatment rankings are based on the existing studies in an NMA; they may not be directly used to inform treatment rankings in future studies due to potential heterogeneity. Heterogeneity is a critical factor in meta-analyses. It is usually quantified by the *I*^2^ statistic, which is interpreted as the percentage of variability due to between-study heterogeneity rather than within-study sampling error [15]. Nevertheless, *I*^2^ has several limitations [16]. For example, researchers commonly report *I*^2^ without an interval estimate quantifying its uncertainty and may wrongly use it as an absolute measure [17]. Motivated by these limitations, the prediction interval is recommended for use in meta-analyses (including NMAs) [17–21]. While the conventional confidence or credible interval informs a treatment effect’s uncertainty based on the studies in a *current* NMA, the prediction interval gives a range of the true treatment effect in *future* studies. The prediction interval may be more straightforward than heterogeneity measures such as *τ*^2^ and *I*^2^ in appraising the impact of heterogeneity. It helps clinicians understand the full uncertainties in treatment effects in future studies.

Similar approaches are needed to predict treatment rankings in a future study setting [22–24]. Such attempts have been made under the frequentist framework and can be implemented using Stata commands [13, 25]. The predictive treatment ranking measures account for the heterogeneity between the future study and the studies in the existing NMA; they may provide important information for future clinical trial designs in the presence of many competing treatment options. For example, for ethical considerations, clinicians might want to compare a new drug with existing treatments that have relatively high predictive measures.

In the current literature, many NMAs are performed using Bayesian approaches alongside frequentist ones [26–28]. Bayesian approaches offer additional flexibility compared to frequentist approaches, e.g., by specifying informative priors and sophisticated variance-covariance structures within multi-arm studies [29–36]. This article extends the P-score to a future study setting under the Bayesian framework, with the focus on NMAs with binary outcomes.

## Methods

### Bayesian model for network meta-analysis

We assume that an NMA contains *N* studies; each study compares a subset of a total of *K* treatments. The treatments compared in study *i* are denoted by the set ${\mathcal{T}}_i$ (*i* = 1, …, *N*). Let *y*_*ik*_ be the outcome measure in study *i*’s treatment group *k* (*k* ∈ *T*_*i*_) and ${\mathcal{D}}_{ik}$ be the known, observed data in this study’s treatment group.

This article focuses on the case of binary outcomes, so *y*_*ik*_ is the event count, which is assumed to follow the binomial distribution, and ${\mathcal{D}}_{ik}$ represents the sample size *n*_*ik*_. Without loss of generality, the following materials can be generalized to other types of outcomes. In addition, let *b*_*i*_ be the baseline treatment for study *i*; this can be any treatment in ${\mathcal{T}}_i$, and can differ across studies. For simplicity, we denote it by *b* when it does not lead to confusion. The Bayesian hierarchical model for the NMA is [2, 37]:

(likelihood)

$${y}_{ik}\sim Bin\left({p}_{ik},{n}_{ik}\right),i=1,\dots, N,k\in {\mathcal{T}}_i;$$

(link function)

$$g\left({p}_{ik}\right)={\mu}_i+{\delta}_{ibk}I\left(k\ne b\right);$$

(random effects)

$${\delta}_{ibk}\sim N\left({d}_{bk},{\tau}_{bk}^2\right);$$

(multi-arm studies)

$$\mathrm{Corr}\left({\delta}_{ibk},{\delta}_{ibh}\right)={\gamma}_{bhk};$$

(priors)

$${\mu}_i,{d}_{bk},{\tau}_{bk},\mathrm{and}\ {\gamma}_{bhk}\sim \mathrm{priors}.$$

We use the canonical logit link function for binomial data, i.e., *g*(*t*) = log[*t*/(1 − *t*)], which transforms the underlying true event rate *p*_*ik*_ to a linear form. The indicator function *I* (·) returns 0 if *k* = *b* and 1 if *k* ≠ *b*. Consequently, *δ*_*ibk*_ represents the underlying true log odds ratio (OR) of treatment *k* vs. *b* in study *i*. Moreover, *μ*_*i*_ represents the baseline effect of study *i*. Because baseline effects could differ greatly across studies, *μ*_*i*_ is commonly modeled as a nuisance parameter. To account for potential heterogeneity, *δ*_*ibk*_ is modeled as a random effect; ${\tau}_{bk}^2$ represents the between-study variance for the comparison *k* vs. *b*. The between-study variances are typically assumed equal for all comparisons (i.e., ${\tau}_{bk}^2={\tau}^2$) [1, 2]. This assumption greatly reduces the model complexity. In cases that these variances dramatically differ, one may alter to use more general model specifications [37]. The overall log ORs are the parameters *d*_*bk*_ and are of primary interest in NMAs. Within multi-arm studies (if any), *γ*_*bhk*_ denotes the correlation coefficient between comparisons *k* vs. *b* and *h* vs. *b*. It is commonly set to 0.5, which is a result of assuming equal between-study variances [2, 38].

The posterior distributions of the parameters of interest can be obtained via the Markov chain Monte Carlo (MCMC) algorithm. Our analyses use the vague priors *N*(0, 100^2^) for study-specific baseline effects *μ*_*i*_ and the log ORs of all treatments vs. the reference treatment, say treatment 1 (i.e., *d*_1*k*_). The reference treatment may be different from study-specific baseline treatments; it is usually a standard control (e.g., placebo) [39]. The overall log ORs of other comparisons are obtained under the evidence consistency assumption, i.e., *d*_*hk*_ = *d*_1*k*_ − *d*_1*h*_ [2]. This article makes the consistency assumption, while it should be routinely checked in NMAs [40–42].

### Frequentist and Bayesian P-scores

We tentatively assume that the outcome is beneficial (e.g., successful smoking cessation); if the outcome is harmful (e.g., mortality), we may simply invert the direction of treatment comparisons in the following materials. Under the frequentist setting, the P-score is built on the quantities

1 $${P}_{kh}=\Phi \left(\left({\hat{d}}_{1k}-{\hat{d}}_{1h}\right)/{s}_{kh}\right),$$

where ${\hat{d}}_{1k}$ and ${\hat{d}}_{1h}$ are the point estimates of treatment effects for *k* vs. 1 and *h* vs. 1, respectively, and *s*_*kh*_ is the standard error of ${\hat{d}}_{1k}-{\hat{d}}_{1h}$. Moreover, Φ(·) is the cumulative distribution function of the standard normal distribution. The quantity *P*_*kh*_ can be interpreted as the extent of certainty that treatment *k* is better than *h* [14]. The *frequentist P-score* of treatment *k* is calculated as $\frac{1}{K-1}{\sum}_{h\ne k}{P}_{kh}$.

Analogously to the frequentist P-score, conditional on *d*_1*h*_ and *d*_1*k*_, the quantities *P*_*kh*_ from the Bayesian perspective can be considered as *I*(*d*_1*k*_ > *d*_1*h*_), which are Bernoulli random variables. To quantify the overall performance of treatment *k*, we may similarly use

2 $${\overline{P}}_k=\frac{1}{K-1}{\sum}_{h\ne k}I\left({d}_{1k}>{d}_{1h}\right).$$

Note that ${\overline{P}}_k$ is a parameter under the Bayesian framework, while the frequentist P-score is a statistic. Moreover, ∑_*h* ≠ *k*_*I*(*d*_1*k*_ > *d*_1*h*_) is equivalent to *K* − *R*_*k*_, where *R*_*k*_ is the true rank of treatment *k*. Thus, we may also write ${\overline{P}}_k=\left(K-{R}_k\right)/\left(K-1\right)$; this corresponds to the findings by Rücker and Schwarzer [14]. Consequently, we call ${\overline{P}}_k$ the *scaled rank in the NMA* for treatment *k*. It transforms the range of the original rank between 1 and *K* to a range between 0 and 1. In addition, note that *E*[*I*(*d*_1*k*_ > *d*_1*h*_| Data)] = Pr(*d*_1*k*_ > *d*_1*h*_| Data), which is analogous to the quantity in Eq. (1) under the frequentist framework. Therefore, we use the posterior mean of the scaled rank ${\overline{P}}_k$ as the *Bayesian P-score*; it is a counterpart of the frequentist P-score.

The scaled ranks ${\overline{P}}_k$ can be feasibly estimated via the MCMC algorithm. Let ${\left\{{d}_{1k}^{(j)};k=2,\dots, K\right\}}_{j=1}^J$ be the posterior samples of the overall relative effects *d*_1*k*_ of all treatments vs. the reference treatment 1 in a total of *J* MCMC iterations after the burn-in period, where *j* indexes the iterations. As *d*_11_ is trivially 0, we set ${d}_{11}^{(j)}$ to 0 for all *j*. The *j*th posterior sample of treatment *k*’s scaled rank is ${\overline{P}}_k^{(j)}=\frac{1}{K-1}{\sum}_{h\ne k}I\left({d}_{1k}^{(j)}>{d}_{1h}^{(j)}\right)$. We can make inferences for the scaled ranks from the posterior samples ${\left\{{\overline{P}}_k^{(j)}\right\}}_{j=1}^J$, and use their posterior means as the Bayesian P-scores. We may also obtain the posterior medians as another set of point estimates, and the 2.5 and 97.5% posterior quantiles as the lower and upper bounds of 95% credible intervals (CrIs), respectively. Because the posterior samples of the scaled ranks take discrete values, the posterior medians and the CrI bounds are also discrete.

### Predictive P-score

Based on the idea of the Bayesian P-score, we can similarly define the predictive P-score for a future study by accounting for the heterogeneity between the existing studies in the NMA and the new study. Specifically, we consider the probabilities in the new study

3 $${P}_{\mathrm{new}, \it{kh}}=\Pr \left({\delta}_{\mathrm{new},1\it {k}}>{\delta}_{\mathrm{new},1\it {h}}\right),$$

conditional on the population parameters *d*_1*h*_, *d*_1*k*_, and *τ* from the NMA. Here, *δ*_new, 1*k*_ and *δ*_new, 1*h*_ represent the treatment effects of *k* vs. 1 and *h* vs. 1 in the new study, respectively. The *P*_new, *kh*_ corresponds to the quantity *P*_*kh*_ in the NMA; it represents the probability of treatment *k* being better than *h* in the new study. Due to heterogeneity, *δ*_new, 1*k*_ ∼ *N*(*d*_1*k*_, *τ*^2^) and *δ*_new, 1*h*_ ∼ *N*(*d*_1*h*_, *τ*^2^). Recall that the correlation coefficients between treatment comparisons are assumed to be 0.5; therefore, such probabilities in the new study can be explicitly calculated as *P*_new, *kh*_ = Φ((*d*_1*k*_ − *d*_1*h*_)/*τ*), which is a function of *d*_1*h*_, *d*_1*k*_, and *τ*. Finally, we use

4 $${\overline{P}}_{\mathrm{new},\it{k}}=\frac{1}{K-1}{\sum}_{h\ne k}{P}_{\mathrm{new}, \it{kh}}$$

to quantify the performance of treatment *k* in the new study. The posterior samples of ${\overline{P}}_{\mathrm{new},k}$ can be derived from the posterior samples of *d*_1*k*_, *d*_1*h*_, and *τ* during the MCMC algorithm.

Note that the probabilities in Eq. (3) can be written as *E*[*I*(*δ*_new, 1*k*_ > *δ*_new, 1*h*_)]. Based on similar observations for the scaled ranks in the NMA, the ${\overline{P}}_{\mathrm{new},k}$ in the new study subsequently becomes

$${\overline{P}}_{\mathrm{new},k}=\frac{1}{K-1}E\left[{\sum}_{h\ne k}I\left({\delta}_{\mathrm{new},1k}>{\delta}_{\mathrm{new},1h}\right)\right]=E\left[\frac{K-{R}_{\mathrm{new},k}}{K-1}\right],$$

where *R*_new, *k*_ is the true rank of treatment *k* in the new study. Thus, we call ${\overline{P}}_{\mathrm{new},\it{k}}$  *the expected scaled rank in the new study*. Like the Bayesian P-score, we define the *predictive P-score* as the posterior mean of ${\overline{P}}_{\mathrm{new},\it{k}}$. The posterior medians and 95% CrIs can also be obtained using the MCMC samples of ${\overline{P}}_{\mathrm{new},\it {k}}$.

Of note, the predictive P-scores considered in this article are all derived under the Bayesian framework. Strictly speaking, they may be called Bayesian predictive P-scores, as contrasted with Bayesian P-scores. Nevertheless, for convenience, we call them predictive P-scores in short.

When *τ* decreases toward 0 (i.e., the fixed-effects setting where all studies share common treatment effects), *P*_new, *kh*_ converges to 0 if *d*_1*k*_ < *d*_1*h*_ or 1 if *d*_1*k*_ > *d*_1*h*_; that is, *P*_new, *kh*_ becomes *I*(*d*_1*k*_ > *d*_1*h*_). Therefore, the expected scaled rank in the new study ${\overline{P}}_{\mathrm{new},k}$ converges to the scaled rank in the NMA ${\overline{P}}_k$, and thus the predictive P-score converges to the Bayesian P-score. Conversely, when *τ* increases toward infinity, *P*_new, *kh*_ converges to 0.5 for all comparisons, so ${\overline{P}}_{\mathrm{new},k}$ (and thus the predictive P-score) converges to 0.5 for each treatment, representing a middle rank. This is consistent with the intuition that the NMA does not provide much information for the new study in the presence of large heterogeneity, as the treatment rankings in the new study are dominated by between-study variabilities.

### Two examples

We give two examples of NMAs with binary outcomes to illustrate the different versions of the P-score. The first example is from Lu and Ades [40]; it was initially reported by Hasselblad [43] (without performing a formal NMA). It investigated the effects of four treatments on smoking cessation, including 1) no contact; 2) self-help; 3) individual counseling; and 4) group counseling. The outcome was the smoking cessation status of an individual after treatment. In the original NMA, the authors found that group counseling was most effective for smoking cessation, followed by individual counseling and self-help, and no contact was the least effective. The dataset contained a total of 16,737 subjects and 24 studies. A treatment was better if it yielded a higher rate of smoking cessation.

The second example was reported by Xu et al. [44]. It investigated the effects of seven immune checkpoint inhibitor (ICI) drugs on all-grade treatment-related adverse events (TrAEs), and aimed to provide a safety ranking of the ICI drugs for the treatment of cancer. The NMA was limited to phase II/III randomized controlled trials that compared two or three of the following treatments: 1) conventional therapy; 2) nivolumab; 3) pembrolizumab; 4) two ICIs; 5) ICI and conventional therapy; 6) atezolizumab; and 7) ipilimumab. The primary outcome was whether the patient had a TrAE. The authors found that there were clinically significant differences in safety between ICI drugs for patients with cancer. In general, atezolizumab was the safest drug, defined by the total number of severe or life-threatening adverse events, followed by nivolumab, pembrolizumab, ipilimumab, and tremelimumab; taking one ICI was found to be safer than taking two ICIs. The dataset contained a total of 126,621 subjects from 23 studies. Unlike the direction in the first example, a treatment was better if it yields a lower rate of TrAEs.

In the following analyses, we will use the numerical labels above to refer to treatments. Appendix A in Additional file 1 gives the complete datasets of the two examples.

### Implementations

The Bayesian NMAs were implemented via the MCMC algorithm using JAGS (version 4.3.0) through the R (version 3.6.2) package “rjags” (version 4–10). We used the vague priors *U*(0, 5) for the heterogeneity standard deviation (SD). We obtained the posterior samples of the log ORs of all treatment comparisons, which were then used to derive the posterior distributions of the Bayesian P-scores and predictive P-scores.

In addition to the vague priors, secondary analyses were performed for each NMA using informative priors [32, 36]. Specifically, based on the recommendations from Turner et al. [32], we used the log-normal priors *LN*(−2.01, 1.64^2^) and *LN*(−2.13, 1.58^2^) for the heterogeneity variances in the smoking cessation and all-grade TrAEs data, respectively.

For each NMA, we used three Markov chains; each chain contained a 20,000-run burn-in period for achieving stabilization and convergence. The final posterior samples consisted of a run of 50,000 updates after the burn-in period with thinning rate 2. We examined the stabilization and convergence of MCMC using trace plots and the Gelman–Rubin convergence statistics $\hat{R}$ of log ORs and the heterogeneity SD [45]. The $\hat{R}$ values close to 1 indicate adequate convergence.

We used the posterior samples to form the posterior distributions and calculate the posterior means (i.e., Bayesian and predictive P-scores), posterior medians, and 95% CrIs for all treatments’ scaled ranks in the NMA and expected scaled ranks in a new study. Additionally, we calculated the frequentist P-scores using the R package “netmeta” (version 1.2.0). The code for all analyses is in Appendix B in Additional file 1.

## Results

Tables 1 and 2 present the treatment ranking measures in the examples of smoking cessation and all-grade TrAEs, respectively. Appendix C in Additional file 1 presents the trace plots. The MCMC iterations stabilized and converged well in both examples; all values of $\hat{R}$ were approximately equal to 1. Appendix D in Additional file 1 presents the treatment ranking measures in the secondary analyses using the informative priors. In the two examples, the informative priors produced similar treatment ranking measures to the vague priors.

**Table 1:** Treatment ranking measures in the example of smoking cessation

Treatment	Mean (P-score)	Median	95% credible interval
Frequentist P-Score:			
1	0.048	NA	NA
2	0.404	NA	NA
3	0.710	NA	NA
4	0.838	NA	NA
Scaled rank in the NMA:			
1	0.038	0.000	(0.000, 0.333)
2	0.394	0.333	(0.000, 1.000)
3	0.689	0.667	(0.333, 1.000)
4	0.879	1.000	(0.333, 1.000)
Expected scaled rank in a new study:			
1	0.192	0.182	(0.061, 0.379)
2	0.440	0.435	(0.189, 0.719)
3	0.623	0.624	(0.425, 0.813)
4	0.746	0.762	(0.456, 0.943)

Note: NA, not applicable. The posterior means of the scaled ranks in the NMA are the Bayesian P-scores, and those of the expected scaled ranks in a new study are the predictive P-scores

**Table 2:** Treatment ranking measures in the example of all-grade treatment-related adverse events

Treatment	Mean (P-score)	Median	95% credible interval
Frequentist P-score:			
1	0.365	NA	NA
2	0.821	NA	NA
3	0.677	NA	NA
4	0.174	NA	NA
5	0.096	NA	NA
6	0.944	NA	NA
7	0.432	NA	NA
Scaled rank in the NMA:			
1	0.362	0.333	(0.167, 0.500)
2	0.764	0.667	(0.667, 1.000)
3	0.780	0.833	(0.500, 1.000)
4	0.164	0.167	(0.000, 0.500)
5	0.092	0.000	(0.000, 0.333)
6	0.924	1.000	(0.667, 1.000)
7	0.415	0.500	(0.000, 0.833)
Expected scaled rank in a new study:			
1	0.360	0.365	(0.190, 0.509)
2	0.748	0.749	(0.588, 0.897)
3	0.757	0.771	(0.488, 0.943)
4	0.202	0.174	(0.008, 0.573)
5	0.141	0.124	(0.009, 0.369)
6	0.873	0.897	(0.613, 0.993)
7	0.418	0.413	(0.155, 0.729)

Note: NA, not applicable. The posterior means of the scaled ranks in the NMA are the Bayesian P-scores, and those of the expected scaled ranks in a new study are the predictive P-scores

The posterior means (Bayesian P-scores) and posterior medians of scaled ranks in the NMAs differed noticeably for both examples. Because the posterior samples of scaled ranks were discrete, as suggested by Eq. (2), the posterior medians took discrete values, while the posterior means (Bayesian P-scores) took continuous values. Due to heterogeneity, the expected scaled ranks in a new study were based on probabilities that could continuously range from 0 to 1 as in Eq. (4); thus, both their posterior means (predictive P-scores) and posterior medians took continuous values.

### Example of smoking cessation

In the example of smoking cessation, Table 1 shows that treatment 4 had the highest Bayesian P-score and thus was likely the best treatment, followed by treatments 3 and 2. Treatment 1 was likely the worst because its Bayesian P-score, 0.038, was closest to 0. The Bayesian P-scores and frequentist P-scores slightly differed; their differences were up to 0.041. Their orders of treatment rankings were identical.

The order of treatment rankings based on the predictive P-scores for the new study also remained consistent with that based on the P-scores for the NMA. Treatment 4 continued to have both the highest P-score in the new study, followed by treatments 3 and 2, and treatment 1 had the lowest value. Compared with P-scores, the predictive P-scores of all four treatments tended to shrink toward 0.5 due to the heterogeneity. For example, the Bayesian P-score of treatment 1 increased from 0.038 to the predictive P-score of 0.192, while the Bayesian P-score of treatment 4 decreased from 0.879 to the predictive P-score of 0.746.

### Example of treatment-related adverse event

In the example of all-grade TrAEs, Table 2 shows that treatment 6 had the highest Bayesian P-score and was thus likely the best treatment. It was followed by treatments 3 and 2 with very similar Bayesian P-scores (0.780 and 0.764, respectively), treatments 7 and 1 also with similar Bayesian P-scores (0.415 and 0.362, respectively), then treatment 4 with a Bayesian P-score of 0.164. Treatment 5 was likely the worst with a Bayesian P-score of 0.092.

Some frequentist P-scores were noticeably different from their Bayesian counterparts. The Bayesian P-score of treatment 2 was 0.764, and its frequentist P-score was 0.821; such P-scores of treatment 3 were 0.780 and 0.677, accordingly. Based on the Bayesian P-scores, treatment 2 was worse than treatment 3, but their rankings were reversed based on the frequentist P-scores. These differences were likely because the Bayesian and frequentist P-scores were derived using different models. The Bayesian model accounted for full uncertainties by modeling event counts with binomial likelihoods, while the frequentist model approximated the log OR of each treatment comparison to the normal distribution within each study.

Compared with the P-scores, the predictive P-scores of most treatments did not change much, likely because the heterogeneity was relatively small in this NMA. As in the example of smoking cessation, the predictive P-scores had the trend of shrinking toward 0.5.

### Visualizations

**Fig. 1:**
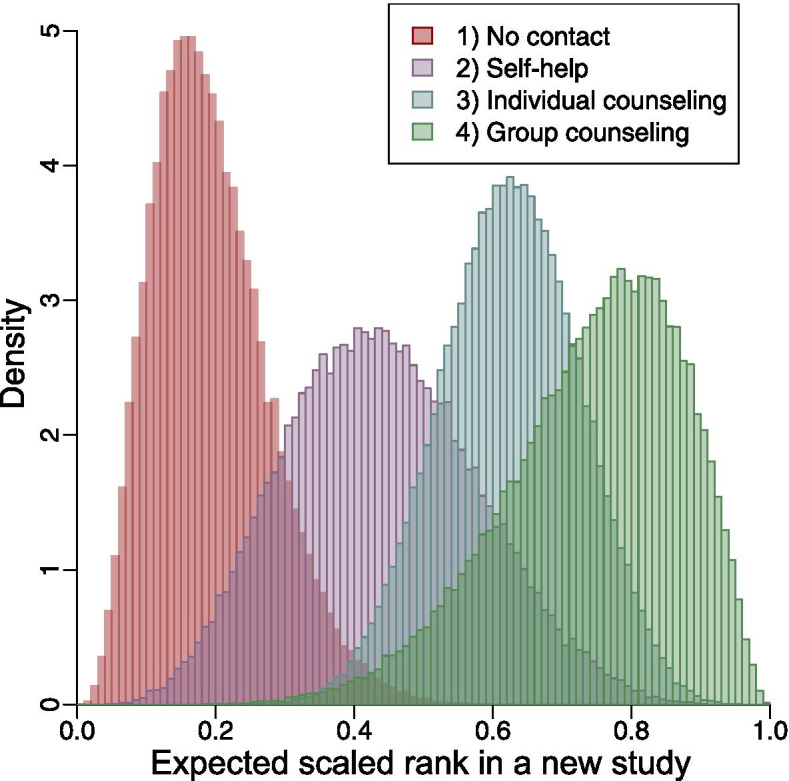
Posterior distributions of all treatments’ expected scaled ranks in a new study in the example of smoking cessation

**Fig. 2:**
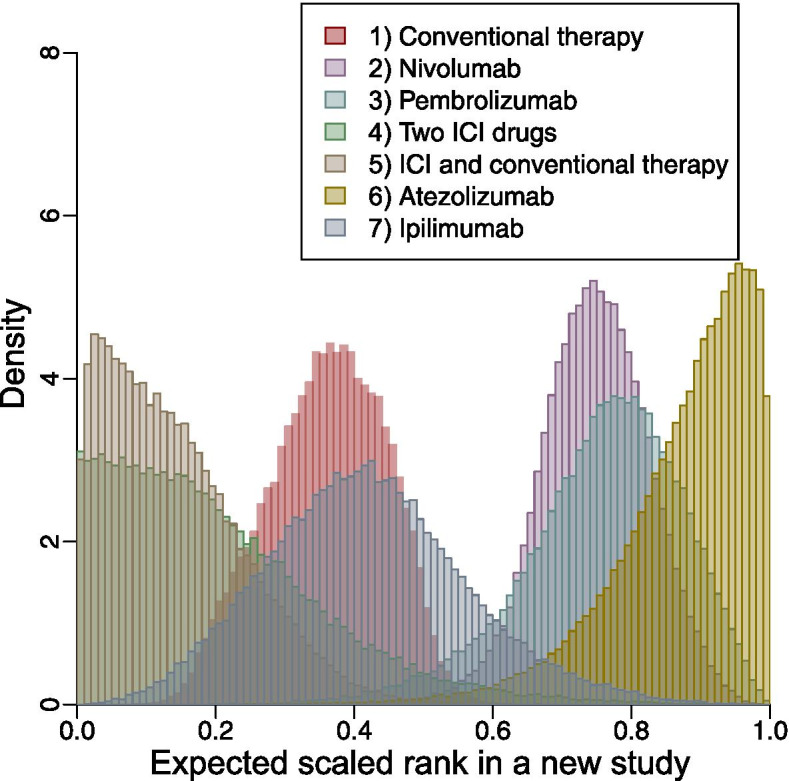
Posterior distributions of all treatments’ expected scaled ranks in a new study in the example of all-grade treatment-related adverse events

Figures 1 and 2 present the posterior distributions of all treatments’ expected scaled ranks in a new study in both examples, where each bar in the histograms covers a range of 0.01. They offer an intuitive tool to compare all treatments simultaneously.

The differences between the posterior means (predictive P-scores) and posterior medians of the expected scaled ranks were relevant to the symmetry of the corresponding posterior distributions. For all treatments in both examples, the posterior distributions were unimodal. The posterior distributions for all treatments in the example of smoking cessation were roughly symmetric, so their posterior medians (predictive P-scores) were close to their posterior means (Table 1). For the example of all-grade TrAEs, the posterior distributions of treatments 4, 5, and 6 were markedly asymmetric; the posterior means (predictive P-scores) of these three treatments were noticeably different from their posterior medians (Table 2). The distributions for other treatments were approximately symmetric, and their posterior means and posterior medians were nearly identical.

## Discussion

### Implications

While the order of treatment rankings in the two examples remained mostly the same, there were noticeable differences between the frequentist P-scores and Bayesian P-scores for some treatments, primarily owing to the different specifications of the frequentist and Bayesian NMA models. In addition, there were possibly discrepancies between posterior means (predictive P-scores) and posterior medians of the expected scaled ranks in a new study (as well as for scaled ranks in the NMA that lead to Bayesian P-scores). These discrepancies depended on the symmetry of the posterior distributions. Though the posterior medians are conventionally used in Bayesian (network) meta-analyses, we used the posterior means for the Bayesian and predictive P-scores because they correspond to the original definition of the P-score under the frequentist framework [14]. In practice, both the posterior mean and posterior median may be reported for measuring treatment rankings.

Like the conventional frequentist P-scores, the predictive P-scores should be interpreted with caution, and their uncertainties ought to be taken into considerations [46, 47]. The magnitudes of the predictive P-scores do not imply statistically significant differences between treatments. Instead of being designed to test for treatment differences, they are treatment-specific summary scores that facilitate clinical interpretations in comparative effectiveness research. Therefore, in addition to the magnitudes of treatment ranking measures, researchers should also pay attention to their uncertainties [46]. The uncertainties can be reflected by the measures’ confidence intervals or CrIs. A benefit of the predictive P-scores is that their CrIs’ limits take continuous values. The CrIs’ limits of the conventional P-scores must take discrete values, which may not accurately reflect the uncertainties. Recently, Wu et al. [48] proposed the normalized entropy to quantify the uncertainties of SUCRA. Similar ideas may be used to measure the predictive P-score’s uncertainties.

### Limitations

This study had several limitations. We have focused on NMAs with binary outcomes and used the OR as the effect measure. The predictive P-score can be applied to generic NMAs by modifying the likelihoods of outcome measures in the Bayesian hierarchical model. Moreover, we have used the Bayesian NMA model that assumes evidence consistency and used a common heterogeneity variance *τ*^2^ for all treatment comparisons. In practice, these assumptions should be carefully examined before applying the predictive P-score to clinical decision-making [37, 49, 50].

### Future directions

This study used two examples to illustrate the predictive P-scores; however, it is unclear how the predictive P-scores might differ from the conventional P-scores in broader applications of NMAs. As a future topic, it is worthwhile to empirically investigate the magnitudes and directions of their changes via a comprehensive collection of NMAs.

## Conclusions

This article has proposed the predictive P-score by extending the Bayesian P-score to the future study setting. The predictive P-score accounts for heterogeneity between the new study and the existing studies in an NMA. It can be used to select optimal treatments from a potentially large pool of options for new patients in the future.

## Supplementary Information

**Additional file 1: Appendix A**. Complete datasets. **Appendix B**. R code for data analyses. **Appendix C**. Trace plots. **Appendix D**. Secondary analyses.

## Data Availability

The Supplementary Information includes the code and data for the analyses presented in this article.

## Abbreviations

CrI: Credible interval
IC: Immune checkpoint inhibitor
MCMC: Markov chain Monte Carlo
NMA: Network meta-analysis
OR: Odds ratio
SD: Standard deviation
SUCRA: Surface under the cumulative ranking curve
TrAE: Treatment-related adverse event
