# Supplementary material for: Predictive P-score for treatment ranking in Bayesian network meta-analysis
Source: BMC Med Res Methodol. 2021 Oct 17;21:213. doi: 10.1186/s12874-021-01397-5 (PMC8520624; doi:10.1186/s12874-021-01397-5)
Supplement: Supplementary file 1 — Additional file 1: Appendix A. Complete datasets. Appendix B. R code for data analyses. Appendix C. Trace plots. Appendix D. Secondary analyses. [file 12874_2021_1397_MOESM1_ESM.pdf]

Supplementary Information for
"Predictive P-score for treatment ranking in Bayesian network meta-analysis"

List of contents

Appendix A. Complete datasets

Appendix B. R code for data analyses

Appendix C. Trace plots

Appendix D. Secondary analyses

Appendix A. Complete datasets

Table S1. Full dataset for the network meta-analysis on smoking cessation

Study ID	Treatment ID	Event count	Sample size
1	1	9	140
1	3	23	140
1	4	10	138
2	2	11	78
2	3	12	85
2	4	29	170
3	1	75	731
3	3	363	714
4	1	2	106
4	3	9	205
5	1	58	549
5	3	237	1561
6	1	0	33
6	3	9	48
7	1	3	100
7	3	31	98
8	1	1	31
8	3	26	95
9	1	6	39
9	3	17	77
10	1	79	702
10	2	77	694
11	1	18	671
11	2	21	535
12	1	64	642
12	3	107	761
13	1	5	62
13	3	8	90
14	1	20	234
14	3	34	237
15	1	0	20
15	4	9	20
16	1	8	116
16	2	19	146
17	1	95	1107
17	3	134	1031
18	1	15	187
18	3	35	504
19	1	78	584
19	3	73	675
20	1	69	1177
20	3	54	888
21	2	20	49
21	3	16	43
22	2	7	66
22	4	32	127
23	3	12	76
23	4	20	74
24	3	9	55
24	4	3	26

Table S2. Full dataset for the network meta-analysis on all-grade treatment-related adverse events

Study ID	Treatment ID	Event count	Sample size
1	1	199	287
1	2	236	268
2	1	76	131
2	2	111	129
3	1	190	267
3	2	243	263
4	1	139	236
4	2	86	111
5	1	206	268
5	2	84	102
6	1	319	406
6	2	349	397
7	1	153	206
7	2	155	205
8	3	248	266
8	2	250	255
9	3	441	682
9	2	251	309
10	3	113	154
10	2	135	150
11	3	252	357
11	2	138	171
12	6	95	142
12	2	119	135
13	6	319	459
13	2	395	443
14	6	390	609
14	2	496	578
15	5	344	388
15	2	292	361
16	5	54	59
16	2	54	62
17	5	109	138
17	2	52	65
18	5	391	478
18	2	361	476
19	5	75	84
19	2	40	44
20	5	192	247
20	2	96	251
21	1	385	452
21	7	434	453
22	1	270	313
22	7	268	311
22	4	300	313
23	4	86	94
23	7	43	46

Appendix B. R code for data analyses

```
## load libraries
library("rjags")
library("coda")
## set your working directory
setwd("...")

############## Functions
## JAGS code for performing NMA

BayesianNMAModel <- function(o){
  out <- "
  model{
  for(i in 1:NS){
  w[i,1] <- 0
  delta[i,t[i,1]] <- 0
  mu[i] ~ dnorm(0, 0.0001)  #vague priors for trial baselines
  for(k in 1:na[i]){
  r[i,k] ~ dbin(p[i,t[i,k]], n[i,k])  #binomial likelihood
  logit(p[i,t[i,k]]) <- mu[i] + delta[i,t[i,k]]  #model
  }
  for(k in 2:na[i]){
  # trial-specific log OR
  delta[i,t[i,k]] ~ dnorm(md[i,t[i,k]], taud[i,t[i,k]])
  md[i,t[i,k]] <- d[t[i,k]] - d[t[i,1]] + sw[i,k]    #mean of LOR
  taud[i,t[i,k]] <- tau*2*(k - 1)/k  #precision of LOR
  # adjustment of multi-arm trials
  w[i,k] <- delta[i,t[i,k]] - d[t[i,k]] + d[t[i,1]]
  sw[i,k] <- sum(w[i,1:(k-1)])/(k - 1)
  }
  }

  d[1] <- 0
  for(k in 2:NT){
  d[k] ~ dnorm(0, 0.0001)
  }

  tau <- pow(sigma, -2)
  sigma ~ dunif(0, 5)  #heterogeneity standard deviation

  # pairwise ORs
  for(h in 1:(NT - 1)){
  for(k in (h + 1):NT){
  lor[h,k] <- d[k] - d[h]
  }
  }

  rank <- rank(d)
  }"
return(out)
}

## P Scores
# dat is a data frame containing the dataset of an NMA
# with binary outcomes. It has four columns:
# sid (study id, from 1 to NS); tid (treatment id, from 1 to NT);
# r (number of reponses); n (sample size).
# n.chains, n.burnin, n.iter, n.thin are the number of chains,
# the number of burnin iterations, the number of iterations after burnin,
# and the thinning rate for the MCMC algorithm.
# lowerbetter = TRUE: lower effect measures indicate better treatments;
# lowerbetter = FALSE: lower effect measures indicate worse treatments
# pred: deriving the treatment ranking measures in a new study
# trace: saving the posterior samples

pscore <- function(dat, n.chains = 3, n.burnin = 2000, n.iter = 5000,
                    thin = 2, lowerbetter = TRUE, pred = TRUE, trace = FALSE){
  NS <- max(dat$sid) # number of studies
  NT <- max(dat$tid) # number of treatments
```

```
na <- as.numeric(table(dat$sid)) # number of arms within study
r <- n <- t <- matrix(NA, nrow = NS, ncol = max(na))
for(i in 1:NS){
  r[i, 1:na[i]] <- dat$r[dat$sid == i]
  n[i, 1:na[i]] <- dat$n[dat$sid == i]
  t[i, 1:na[i]] <- dat$tid[dat$sid == i]
}

jags.dat <- list(NS = NS, NT = NT, na = na, r = r, n = n, t = t)
jags.inits <- list(
  list(mu = rep(0, NS), d = c(NA, rep(0, NT - 1)), sigma = 0.5),
  list(mu = rep(0.5, NS), d = c(NA, rep(-0.5, NT - 1)), sigma = 0.3),
  list(mu = rep(-0.5, NS), d = c(NA, rep(0.5, NT - 1)), sigma = 0.8))
jags.m <- jags.model(file = textConnection(BayesianNMAModel()),
                     data = jags.dat, inits = jags.inits,
                     n.chains = n.chains, n.adapt = 1000)
update(jags.m, n.iter = n.burnin)
params <- c("d","sigma")
coda <- coda.samples(model = jags.m, variable.names = params,
                     n.iter = n.iter, thin = thin)

all.post <- NULL
for(i in 1:n.chains){
  all.post <- rbind(all.post, coda[[i]])
}
all.post <- t(all.post)


iter.post <- dim(all.post)[2]
d.post <- all.post[paste0("d[", 1:NT, "]"),]
dim <- ncol(d.post)
all.trts <- 1:NT


#P-Score
P <- rep(list(NULL), NT)
for(k in 1:NT){
  x <- (matrix(rep(d.post[k,], NT - 1), byrow = TRUE, nrow = NT - 1)
        - d.post[-k,])
  x <- t(x)
  y <- matrix(NA, nrow = dim, ncol = NT-1)
  yavg <- matrix(NA, nrow = dim, ncol = 1)
  for(p in 1:dim){
    for(q in 1:(NT-1))
      if(!lowerbetter){
        if (x[p,q] > 0) {y[p,q] = 1}
        else(y[p,q] = 0)
      }else {
        if (x[p,q] < 0) {y[p,q] = 1}
        else(y[p,q] = 0)
      }
    yavg[p] = mean(y[p,])
  }
  P[[k]] <- yavg
}


P.score <-  matrix(NA, nrow = NT, ncol = 4)
colnames(P.score) <-
  c("Mean", "Median", "95% CrI LB", "95% CrI UB")
for(k in 1:NT){
  P.score[k,1] <- mean(P[[k]])
  P.score[k, 2:4] <- as.numeric(quantile(P[[k]],
                                         probs = c(0.5, 0.025, 0.975)))
}

  if(pred){
    if(!lowerbetter) d.post <- -d.post
    sigma.post <- all.post["sigma",]

    P.pred <- rep(list(NULL), NT)
```

```
      for(k in 1:NT){
        x <-
          (matrix(rep(d.post[k,], NT - 1), byrow = TRUE, nrow = NT - 1) -
             d.post[-k,])/
          matrix(rep(sigma.post, NT - 1), byrow = TRUE, nrow = NT - 1)
        x <- t(x)
        out <- pnorm(x, mean = 0, sd = 1)
        yavg <- matrix(NA, nrow = dim, ncol = 1)
        for(p in 1:dim){
          yavg[p] = mean(out[p,])
        }
        P.pred[[k]] <- yavg
      }

    P.score.pred <- matrix(NA, nrow = NT, ncol = 4)
    colnames(P.score.pred) <-
      c("Mean", "Median", "95% CrI LB", "95% CrI UB")
    for(k in 1:NT){
      P.score.pred[k,1] <- mean(P.pred[[k]])
      P.score.pred[k, 2:4] <- as.numeric(quantile(P.pred[[k]],
                                    probs = c(0.5, 0.025, 0.975)))
    }

    out <- list(P.score = P.score,
                P.pred = P.pred, P.score.pred = P.score.pred)
  }else{
    out <- list(P.score = P.score)
  }

  if(trace) out$coda <- coda
  return(out)
}
## Smoking cessation data
dat.sc <- read.csv("SmokingCessation.csv")

set.seed(1234)
begin <- Sys.time()
pscore.sc <- pscore(dat = dat.sc, n.chains = 3,
                        n.burnin = 20000, n.iter = 50000, thin = 2,
                        lowerbetter = FALSE, pred = TRUE, trace = TRUE)
end <- Sys.time()
end - begin

pscore.sc$P.score

pscore.sc$P.score.pred

#Analysis for Xu 2018 data
dat.xu <- read.csv("Xu 2018.csv")

set.seed(1234)
begin <- Sys.time()
pscore.xu <- pscore(dat = dat.xu, n.chains = 3,
                        n.burnin = 20000, n.iter = 50000, thin = 2,
                        lowerbetter = TRUE, pred = TRUE, trace = TRUE)
end <- Sys.time()
end - begin

pscore.xu$P.score

pscore.xu$P.score.pred

#################################################
# Calculate p-score under frequentist framework
data(smokingcessation)

p1 <- pairwise(treat = list(treat1, treat2, treat3),
               event = list(event1, event2, event3),
               n = list(n1, n2, n3),
               data = smokingcessation, sm = "OR")
```

```
net1 <- netmeta(TE, seTE, treat1, treat2, studlab, data = p2,
                comb.fixed = FALSE, comb.random = TRUE)
summary(net1)

netrank(net1,small.values = "bad")

xu.freq <- read.csv("Xu 2018 freq.csv")

p2 <- pairwise(treat = list(treat1, treat2, treat3),
               event = list(event1, event2, event3),
               n = list(n1, n2, n3),
               data = xu.freq, sm = "OR")

net2 <- netmeta(TE, seTE, treat1, treat2, studlab, data = p2,
                comb.fixed = FALSE, comb.random = TRUE)

summary(net2)

netrank(net2,small.values = "good")


######################################################################
##### PLOTS
## Traceplot for the smoking cessation data

set.seed(1234)
begin <- Sys.time()
out.rankprob <- pscore(dat = dat.sc, n.chains = 3,
                       n.burnin = 20000, n.iter = 100000, thin = 2,
                       lowerbetter = FALSE, pred = TRUE, trace = TRUE)
end <- Sys.time()
end - begin

NT <- 4
for(k in 2:NT){
  png(paste0("SmokingCessationTraceplotd1", k, ".png"),
      res = 600, height = 8.5, width = 11, units = "in")
  par(mar = c(3.5, 4, 2, 1) + 0.1,
      mfrow = c(length(pscore.sc$coda), 1))
  temp <- NULL
  for(i in 1:length(pscore.sc$coda)){
    temp <- c(temp,
              as.vector(pscore.sc$coda[[i]][,paste0("d[", k, "]")]))
  }
  y.range <- range(temp)
  for(i in 1:length(pscore.sc$coda)){
    temp <- as.vector(pscore.sc$coda[[i]][,paste0("d[", k, "]")])
    plot(temp, type = "l", col = "red", xlab = "", ylab = "",
         ylim = y.range, cex.axis = 1.2)
    title(xlab = "Iteration", line = 2.2, cex.lab = 2)
    title(ylab = paste("Log OR of", k, "vs. 1"),
          line = 2.2, cex.lab = 2)
    title(main = paste("Chain", i),
          font.main = 1, cex.main = 2, line = 0.5)
  }
  dev.off()
}

png("SmokingCessationTraceplotsigma.png",
    res = 600, height = 8.5, width = 11, units = "in")
par(mar = c(3.5, 4, 2, 1) + 0.1,
    mfrow = c(length(pscore.sc$coda), 1))
temp <- NULL
for(i in 1:length(pscore.sc$coda)){
  temp <- c(temp,
            as.vector(pscore.sc$coda[[i]][,"sigma"]))
}
y.range <- range(temp)
for(i in 1:length(pscore.sc$coda)){
  temp <- as.vector(pscore.sc$coda[[i]][,"sigma"])
  plot(temp, type = "l", col = "red", xlab = "", ylab = "",
```

```
       ylim = y.range, cex.axis = 1.2)
  title(xlab = "Iteration", line = 2.2, cex.lab = 2)
  title(ylab = "Heterogeneity SD",
        line = 2.2, cex.lab = 2)
  title(main = paste("Chain", i),
        font.main = 1, cex.main = 2, line = 0.5)
}
dev.off()

pdf("SmokingCessationPredPHist.pdf", width = 6, height = 6)
par(mar = c(3, 3, 0.5, 0.8) + 0.1, mgp = c(2.5, 0.5, 0))
NT <- 4
cols <- c("red4", "plum4", "paleturquoise4", "palegreen4")
trt <- c("1) No contact", "2) Self-help", "3) Individual counseling", "4) Group
counseling")cols.hist <- adjustcolor(cols, alpha.f = 0.4)
histp <- list(NULL)
for(k in 1:NT){
  temp <- pscore.sc$P.pred[[k]]
  temp[temp < 0] <- 0
  temp[temp > 1] <- 1
  histp[[k]] <- hist(temp, breaks = seq(0, 1, 0.010), plot = FALSE)
}
max.lim <- max(c(histp[[1]]$density), c(histp[[2]]$density),
               c(histp[[3]]$density), c(histp[[4]]$density))
for(k in 1:NT){
  if(k == 1){
    plot(histp[[k]], col = cols.hist[k], xlim = c(0, 1),
         ylim = c(0, max.lim), freq = FALSE, border = cols.hist[k],
         main = "", xlab = "", ylab = "", xaxs = "i", yaxs = "i",
         cex.axis = 1.2)
    title(xlab = "Expected scaled rank in a new study", line = 1.8, cex.lab = 1.5)
    title(ylab = "Density", line = 1.8, cex.lab = 1.5)
  }else{
    plot(histp[[k]], col = cols.hist[k], xlim = c(0, 1),
         ylim = c(0, max.lim), freq = FALSE, border = cols[k],
         add = TRUE)
  }
}
legend("topright", fill = cols.hist, border = cols,
       legend = trt, cex = 1.2)
dev.off()

# Plots for trAEs data

NT <- 7
for(k in 2:NT){
  png(paste0("trAEsTraceplotd1", k, ".png"),
      res = 600, height = 8.5, width = 11, units = "in")
  par(mar = c(3.5, 4, 2, 1) + 0.1,
      mfrow = c(length(pscore.xu$coda), 1))
  temp <- NULL
  for(i in 1:length(pscore.xu$coda)){
    temp <- c(temp,
              as.vector(pscore.xu$coda[[i]][,paste0("d[", k, "]")]))
  }
  y.range <- range(temp)
  for(i in 1:length(pscore.xu$coda)){
    temp <- as.vector(pscore.xu$coda[[i]][,paste0("d[", k, "]")])
    plot(temp, type = "l", col = "red", xlab = "", ylab = "",
         ylim = y.range, cex.axis = 1.2)
    title(xlab = "Iteration", line = 2.2, cex.lab = 2)
    title(ylab = paste("Log OR of", k, "vs. 1"),
          line = 2.2, cex.lab = 2)
    title(main = paste("Chain", i),
          font.main = 1, cex.main = 2, line = 0.5)
  }
  dev.off()
}

png("trAEsTraceplotsigma.png",
    res = 600, height = 8.5, width = 11, units = "in")
```

```
par(mar = c(3.5, 4, 2, 1) + 0.1,
    mfrow = c(length(pscore.xu$coda), 1))
temp <- NULL
for(i in 1:length(pscore.xu$coda)){
  temp <- c(temp,
            as.vector(pscore.xu$coda[[i]][,"sigma"]))
}
y.range <- range(temp)
for(i in 1:length(pscore.xu$coda)){
  temp <- as.vector(pscore.xu$coda[[i]][,"sigma"])
  plot(temp, type = "l", col = "red", xlab = "", ylab = "",
       ylim = y.range, cex.axis = 1.2)
  title(xlab = "Iteration", line = 2.2, cex.lab = 2)
  title(ylab = "Heterogeneity SD",
        line = 2.2, cex.lab = 2)
  title(main = paste("Chain", i),
        font.main = 1, cex.main = 2, line = 0.5)
}
dev.off()

pdf("trAEsPredPHist.pdf", width = 6, height = 6)
par(mar = c(3, 3, 0.5, 0.8) + 0.1, mgp = c(2.5, 0.5, 0))
NT <- 7
cols <- c("red4", "plum4", "paleturquoise4", "palegreen4",
           "navajowhite4", "gold 4", "lightsteelblue4")
trt <- c("1) Conventional therapy", "2) Nivolumab",
          "3) Pembrolizumab", "4) Two ICI drugs",
          "5) ICI and conventional therapy",
          "6) Atezolizumab", "7) Ipilimumab")
cols.hist <- adjustcolor(cols, alpha.f = 0.4)
histp <- list(NULL)
for(k in 1:NT){
  temp <- pscore.xu$P.pred[[k]]
  temp[temp < 0] <- 0
  temp[temp > 1] <- 1
  histp[[k]] <- hist(temp, breaks = seq(0, 1, 0.010), plot = FALSE)
}
max.lim <- max(c(histp[[1]]$density), c(histp[[2]]$density),
               c(histp[[3]]$density), c(histp[[4]]$density))
for(k in 1:NT){
  if(k == 1){
    plot(histp[[k]], col = cols.hist[k], xlim = c(0, 1),
         ylim = c(0, max.lim), freq = FALSE, border = cols.hist[k],
         main = "", xlab = "", ylab = "", xaxs = "i", yaxs = "i",
         cex.axis = 1.2)
    title(xlab = "Expected scaled rank in a new study", line = 1.8, cex.lab = 1.5)
    title(ylab = "Density", line = 1.8, cex.lab = 1.5)
  }else{
    plot(histp[[k]], col = cols.hist[k], xlim = c(0, 1),
         ylim = c(0, max.lim), freq = FALSE, border = cols[k],
         add = TRUE)
  }
}
legend(“topright”, fill = cols.hist, border = cols,
       legend = trt, cex = 1.2)
dev.off()

################################################################
#Rhat for conversion analysis
## XU
xu1 <- pscore.xu$coda[1]
xu2 <- pscore.xu$coda[2]
xu3 <- pscore.xu$coda[3]

#d1-d7
for(i in 1:7){
  nam <- paste("xu.d", i, sep = "")

  tmp1 <- data.frame(m1 = unlist(xu1[,i]))
  tmp2 <- data.frame(m2 = unlist(xu2[,i]))
  tmp3 <- data.frame(m3 = unlist(xu3[,i]))
```

```
  assign(nam,as.matrix(cbind(tmp1,tmp2,tmp3)))
}

#sigma
tmp1 <- data.frame(m1 = unlist(xu1[,8]))
tmp2 <- data.frame(m2 = unlist(xu2[,8]))
tmp3 <- data.frame(m3 = unlist(xu3[,8]))

xu.sig <- as.matrix(cbind(tmp1,tmp2,tmp3))

R.hat(xu.d2)
R.hat(xu.d3)
R.hat(xu.d4)
R.hat(xu.d5)
R.hat(xu.d6)
R.hat(xu.d7)
R.hat(xu.sig)

## Smoking cessation
sc1 <- pscore.sc$coda[1]
sc2 <- pscore.sc$coda[2]
sc3 <- pscore.sc$coda[3]

#d1-d4
for(i in 1:4){
  nam <- paste("sc.d", i, sep = "")

  tmp1 <- data.frame(m1 = unlist(sc1[,i]))
  tmp2 <- data.frame(m2 = unlist(sc2[,i]))
  tmp3 <- data.frame(m3 = unlist(sc3[,i]))

  assign(nam,as.matrix(cbind(tmp1,tmp2,tmp3)))
}

#sigma
tmp1 <- data.frame(m1 = unlist(sc1[,5]))
tmp2 <- data.frame(m2 = unlist(sc2[,5]))
tmp3 <- data.frame(m3 = unlist(sc3[,5]))

sc.sig <- as.matrix(cbind(tmp1,tmp2,tmp3))

R.hat(sc.d2)
R.hat(sc.d3)
R.hat(sc.d4)
R.hat(sc.sig)

# Informative prior analysis
non_pharm <- function(o){
  out <- "
  model{
  for(i in 1:NS){
  w[i,1] <- 0
  delta[i,t[i,1]] <- 0
  mu[i] ~ dnorm(0,.0001)  # vague priors for trial baselines
  for(k in 1:na[i]){
  r[i,k] ~ dbin(p[i,t[i,k]], n[i,k])  # binomial likelihood
  logit(p[i,t[i,k]]) <- mu[i] + delta[i,t[i,k]]  # model
  }
  for(k in 2:na[i]){
  delta[i,t[i,k]] ~ dnorm(md[i,t[i,k]], taud[i,t[i,k]])  # trial-specific LOR distributions
  md[i,t[i,k]] <- d[t[i,k]] - d[t[i,1]] + sw[i,k]  # mean of LOR distributions
  taud[i,t[i,k]] <- prec*2*(k - 1)/k  # precision of LOR distributions
  w[i,k] <- delta[i,t[i,k]] - d[t[i,k]] + d[t[i,1]]  # adjustment, multi-arm RCTs
  sw[i,k] <- sum(w[i,1:(k-1)])/(k - 1)  # cumulative adjustment for multi-arm trials
  }
  }

  d[1] <- 0
  for(k in 2:NT){
  d[k] ~ dnorm(0, 0.0001)
```

```
  }

  prec <- 1/tau2
  tau2 ~ dlnorm(-2.01, 1/1.64^2)  #  prior for random effects standard deviation

  # pairwise ORs
  for(c in 1:(NT - 1)){
  for(k in (c + 1):NT){
  lor[c,k] <- d[k] - d[c]
  }
  }
  }"
  return(out)
}

pscore <- function(dat, n.chains = 3, n.burnin = 2000, n.iter = 5000,
                   thin = 2, lowerbetter = TRUE, pred = TRUE, trace = FALSE){
  NS <- max(dat$sid) # number of studies
  NT <- max(dat$tid) # number of treatments
  na <- as.numeric(table(dat$sid)) # number of arms within study
  r <- n <- t <- matrix(NA, nrow = NS, ncol = max(na))
  for(i in 1:NS){
    r[i, 1:na[i]] <- dat$r[dat$sid == i]
    n[i, 1:na[i]] <- dat$n[dat$sid == i]
    t[i, 1:na[i]] <- dat$tid[dat$sid == i]
  }

  jags.dat <- list(NS = NS, NT = NT, na = na, r = r, n = n, t = t)
  jags.inits <- list(
    list(mu = rep(0, NS), d = c(NA, rep(0, NT - 1)), sigma = 0.5),
    list(mu = rep(0.5, NS), d = c(NA, rep(-0.5, NT - 1)), sigma = 0.3),
    list(mu = rep(-0.5, NS), d = c(NA, rep(0.5, NT - 1)), sigma = 0.8))
  jags.m <- jags.model(file = textConnection(non_pharm()),
                       data = jags.dat, inits = jags.inits,
                       n.chains = n.chains, n.adapt = 1000)
  update(jags.m, n.iter = n.burnin)
  params <- c("d","sigma")
  coda <- coda.samples(model = jags.m, variable.names = params,
                       n.iter = n.iter, thin = thin)

  all.post <- NULL
  for(i in 1:n.chains){
    all.post <- rbind(all.post, coda[[i]])
  }
  all.post <- t(all.post)


  iter.post <- dim(all.post)[2]
  d.post <- all.post[paste0("d[", 1:NT, "]"),]
  dim <- ncol(d.post)
  all.trts <- 1:NT


  #P-Score
  P <- rep(list(NULL), NT)
  for(k in 1:NT){
    x <- (matrix(rep(d.post[k,], NT - 1), byrow = TRUE, nrow = NT - 1)
          - d.post[-k,])
    x <- t(x)
    y <- matrix(NA, nrow = dim, ncol = NT-1)
    yavg <- matrix(NA, nrow = dim, ncol = 1)
    for(p in 1:dim){
      for(q in 1:(NT-1))
        if(!lowerbetter){
          if (x[p,q] > 0) {y[p,q] = 1}
          else(y[p,q] = 0)
        }else {
          if (x[p,q] < 0) {y[p,q] = 1}
          else(y[p,q] = 0)
        }
      yavg[p] = mean(y[p,])
```

```
    }
    P[[k]] <- yavg
  }


  P.score <-  matrix(NA, nrow = NT, ncol = 4)
  colnames(P.score) <-
    c("Mean", "Median", "95% CrI LB", "95% CrI UB")
  for(k in 1:NT){
    P.score[k,1] <- mean(P[[k]])
    P.score[k, 2:4] <- as.numeric(quantile(P[[k]],
                                           probs = c(0.5, 0.025, 0.975)))
  }

  if(pred){
    if(lowerbetter) d.post <- -d.post
    sigma.post <- all.post["sigma",]

    P.pred <- rep(list(NULL), NT)
    for(k in 1:NT){
      x <-
        (matrix(rep(d.post[k,], NT - 1), byrow = TRUE, nrow = NT - 1) -
           d.post[-k,])/
        matrix(rep(sigma.post, NT - 1), byrow = TRUE, nrow = NT - 1)
      x <- t(x)
      out <- pnorm(x, mean = rep(0, NT - 1), sd = 1)
      yavg <- matrix(NA, nrow = dim, ncol = 1)
      for(p in 1:dim){
        yavg[p] = mean(out[p,])
      }
      P.pred[[k]] <- yavg
    }

    P.score.pred <- matrix(NA, nrow = NT, ncol = 4)
    colnames(P.score.pred) <-
      c("Mean", "Median", "95% CrI LB", "95% CrI UB")
    for(k in 1:NT){
      P.score.pred[k,1] <- mean(P.pred[[k]])
      P.score.pred[k, 2:4] <- as.numeric(quantile(P.pred[[k]],
                                                  probs = c(0.5, 0.025, 0.975)))
    }

    out <- list(P.score = P.score,
                P.pred = P.pred, P.score.pred = P.score.pred)
  }else{
    out <- list(P.score = P.score)
  }

  if(trace) out$coda <- coda
  return(out)
}

set.seed(1234)
begin <- Sys.time()
pscore.sc <- pscore(dat = dat.sc, n.chains = 3,
                    n.burnin = 20000, n.iter = 50000, thin = 2,
                    lowerbetter = FALSE, pred = TRUE, trace = TRUE)

pscore.sc$P.score

end <- Sys.time()
end - begin

########################################################################
# All-grade TRAEs
sub_pharm_cont <- function(o){
  out <- "
  model{
  for(i in 1:NS){
  w[i,1] <- 0
  delta[i,t[i,1]] <- 0
```

```
  mu[i] ~ dnorm(0,.0001)  # vague priors for trial baselines
  for(k in 1:na[i]){
  r[i,k] ~ dbin(p[i,t[i,k]], n[i,k])  # binomial likelihood
  logit(p[i,t[i,k]]) <- mu[i] + delta[i,t[i,k]]  # model
  }
  for(k in 2:na[i]){
  delta[i,t[i,k]] ~ dnorm(md[i,t[i,k]], taud[i,t[i,k]])  # trial-specific LOR distributions
  md[i,t[i,k]] <- d[t[i,k]] - d[t[i,1]] + sw[i,k]  # mean of LOR distributions
  taud[i,t[i,k]] <- prec*2*(k - 1)/k  # precision of LOR distributions
  w[i,k] <- delta[i,t[i,k]] - d[t[i,k]] + d[t[i,1]]  # adjustment, multi-arm RCTs
  sw[i,k] <- sum(w[i,1:(k-1)])/(k - 1)  # cumulative adjustment for multi-arm trials
  }
  }

  d[1] <- 0
  for(k in 2:NT){
  d[k] ~ dnorm(0, 0.0001)
  }

  prec <- 1/tau2
  tau2 ~ dlnorm(-2.13, 1/1.58^2)  #  prior for random effects standard deviation

  # pairwise ORs
  for(c in 1:(NT - 1)){
  for(k in (c + 1):NT){
  lor[c,k] <- d[k] - d[c]
  }
  }
  }"
  return(out)
}

pscore <- function(dat, n.chains = 3, n.burnin = 2000, n.iter = 5000,
                   thin = 2, lowerbetter = TRUE, pred = TRUE, trace = FALSE){
  NS <- max(dat$sid) # number of studies
  NT <- max(dat$tid) # number of treatments
  na <- as.numeric(table(dat$sid)) # number of arms within study
  r <- n <- t <- matrix(NA, nrow = NS, ncol = max(na))
  for(i in 1:NS){
    r[i, 1:na[i]] <- dat$r[dat$sid == i]
    n[i, 1:na[i]] <- dat$n[dat$sid == i]
    t[i, 1:na[i]] <- dat$tid[dat$sid == i]
  }

  jags.dat <- list(NS = NS, NT = NT, na = na, r = r, n = n, t = t)
  jags.inits <- list(
    list(mu = rep(0, NS), d = c(NA, rep(0, NT - 1)), sigma = 0.5),
    list(mu = rep(0.5, NS), d = c(NA, rep(-0.5, NT - 1)), sigma = 0.3),
    list(mu = rep(-0.5, NS), d = c(NA, rep(0.5, NT - 1)), sigma = 0.8))
  jags.m <- jags.model(file = textConnection(sub_pharm_cont()),
                       data = jags.dat, inits = jags.inits,
                       n.chains = n.chains, n.adapt = 1000)
  update(jags.m, n.iter = n.burnin)
  params <- c("d","sigma")
  coda <- coda.samples(model = jags.m, variable.names = params,
                       n.iter = n.iter, thin = thin)

  all.post <- NULL
  for(i in 1:n.chains){
    all.post <- rbind(all.post, coda[[i]])
  }
  all.post <- t(all.post)


  iter.post <- dim(all.post)[2]
  d.post <- all.post[paste0("d[", 1:NT, "]"),]
  dim <- ncol(d.post)
  all.trts <- 1:NT


  #P-Score
```

```
  P <- rep(list(NULL), NT)
  for(k in 1:NT){
    x <- (matrix(rep(d.post[k,], NT - 1), byrow = TRUE, nrow = NT - 1)
          - d.post[-k,])
    x <- t(x)
    y <- matrix(NA, nrow = dim, ncol = NT-1)
    yavg <- matrix(NA, nrow = dim, ncol = 1)
    for(p in 1:dim){
      for(q in 1:(NT-1))
        if(!lowerbetter){
          if (x[p,q] > 0) {y[p,q] = 1}
          else(y[p,q] = 0)
        }else {
          if (x[p,q] < 0) {y[p,q] = 1}
          else(y[p,q] = 0)
        }
      yavg[p] = mean(y[p,])
    }
    P[[k]] <- yavg
  }


  P.score <-  matrix(NA, nrow = NT, ncol = 4)
  colnames(P.score) <-
    c("Mean", "Median", "95% CrI LB", "95% CrI UB")
  for(k in 1:NT){
    P.score[k,1] <- mean(P[[k]])
    P.score[k, 2:4] <- as.numeric(quantile(P[[k]],
                                           probs = c(0.5, 0.025, 0.975)))
  }

  if(pred){
    if(lowerbetter) d.post <- -d.post
    sigma.post <- all.post["sigma",]

    P.pred <- rep(list(NULL), NT)
    for(k in 1:NT){
      x <-
        (matrix(rep(d.post[k,], NT - 1), byrow = TRUE, nrow = NT - 1) -
           d.post[-k,])/
        matrix(rep(sigma.post, NT - 1), byrow = TRUE, nrow = NT - 1)
      x <- t(x)
      out <- pnorm(x, mean = rep(0, NT - 1), sd = 1)
      yavg <- matrix(NA, nrow = dim, ncol = 1)
      for(p in 1:dim){
        yavg[p] = mean(out[p,])
      }
      P.pred[[k]] <- yavg
    }

    P.score.pred <- matrix(NA, nrow = NT, ncol = 4)
    colnames(P.score.pred) <-
      c("Mean", "Median", "95% CrI LB", "95% CrI UB")
    for(k in 1:NT){
      P.score.pred[k,1] <- mean(P.pred[[k]])
      P.score.pred[k, 2:4] <- as.numeric(quantile(P.pred[[k]],
                                                  probs = c(0.5, 0.025, 0.975)))
    }

    out <- list(P.score = P.score,
                P.pred = P.pred, P.score.pred = P.score.pred)
  }else{
    out <- list(P.score = P.score)
  }

  if(trace) out$coda <- coda
  return(out)
}

#Analysis for Xu 2018 data
```

```
set.seed(1234)
begin <- Sys.time()
pscore.xu <- pscore(dat = dat.xu, n.chains = 3,
                    n.burnin = 20000, n.iter = 50000, thin = 2,
                    lowerbetter = TRUE, pred = TRUE, trace = TRUE)
end <- Sys.time()
end - begin

#Predictive SUCRA code, for comparison (adapted from Lin 2019)
#SMOKING CESSATION
trtrank <- function(dat, n.chains = 3, n.burnin = 2000, n.iter = 5000,
                    thin = 2, lowerbetter = TRUE, pred = TRUE, trace = FALSE){
  NS <- max(dat$sid) # number of studies
  NT <- max(dat$tid) # number of treatments
  na <- as.numeric(table(dat$sid)) # number of arms within study
  r <- n <- t <- matrix(NA, nrow = NS, ncol = max(na))
  for(i in 1:NS){
    r[i, 1:na[i]] <- dat$r[dat$sid == i]
    n[i, 1:na[i]] <- dat$n[dat$sid == i]
    t[i, 1:na[i]] <- dat$tid[dat$sid == i]
  }

  jags.dat <- list(NS = NS, NT = NT, na = na, r = r, n = n, t = t)
  jags.inits <- list(
    list(mu = rep(0, NS), d = c(NA, rep(0, NT - 1)), sigma = 0.5),
    list(mu = rep(0.5, NS), d = c(NA, rep(-0.5, NT - 1)), sigma = 0.3),
    list(mu = rep(-0.5, NS), d = c(NA, rep(0.5, NT - 1)), sigma = 0.8))
  jags.m <- jags.model(file = textConnection(non_pharm()),
                       data = jags.dat, inits = jags.inits,
                       n.chains = n.chains, n.adapt = 1000)
  update(jags.m, n.iter = n.burnin)
  params <- c("d", "lor", "sigma", "rank")
  coda <- coda.samples(model = jags.m, variable.names = params,
                       n.iter = n.iter, thin = thin)

  all.post <- NULL
  for(i in 1:n.chains){
    all.post <- rbind(all.post, coda[[i]])
  }
  all.post <- t(all.post)

  rank.post <- all.post[grep(pattern = "rank", x = rownames(all.post)),]
  if(!lowerbetter) rank.post <- NT + 1 - rank.post
  SUCRA.post <- (NT - rank.post)/(NT - 1)
  rank <- SUCRA <- matrix(NA, nrow = NT, ncol = 4)
  colnames(rank) <- colnames(SUCRA) <-
    c("Mean", "Median", "95% CrI LB", "95% CrI UB")
  for(k in 1:NT){
    rank[k, 1] <- mean(rank.post[k,])
    rank[k, 2:4] <- as.numeric(quantile(rank.post[k,],
                                        probs = c(0.5, 0.025, 0.975)))
    SUCRA[k, 1] <- mean(SUCRA.post[k,])
    SUCRA[k, 2:4] <- as.numeric(quantile(SUCRA.post[k,],
                                         probs = c(0.5, 0.025, 0.975)))
  }

  if(pred){
    iter.post <- dim(all.post)[2]
    d.post <- all.post[paste0("d[", 1:NT, "]"),]
    if(!lowerbetter) d.post <- -d.post
    sigma.post <- all.post["sigma",]
    all.trts <- 1:NT
    Psi <- matrix(0.5, NT - 1, NT - 1)
    diag(Psi) <- 1

    pr.rank.new.post <- pr.rank.new.w.post <-
      array(dim = c(NT, NT, iter.post))
    for(k in 1:NT){
      for(r in 1:NT){
        if(r == 1){
          x <-
```

```
          -(matrix(rep(d.post[k,], NT - 1), byrow = TRUE, nrow = NT - 1) -
              d.post[-k,])/
          matrix(rep(sigma.post, NT - 1), byrow = TRUE, nrow = NT - 1)
        x <- t(x)
        out <- pmnorm(x = x, mean = rep(0, NT - 1), varcov = Psi)
      }
      if(r > 1 & r < NT){
        better <- combn(x = all.trts[-k], m = r - 1)
        out <- rep(0, iter.post)
        for(i in 1:dim(better)[2]){
          sgn <- rep(1, NT)
          sgn[-better[,i]] <- -1
          sgn <- sgn[-k]
          x <-
            matrix(rep(sgn, iter.post), byrow = FALSE, nrow = NT - 1)*
            (matrix(rep(d.post[k,], NT - 1), byrow = TRUE, nrow = NT - 1) -
               d.post[-k,])/
            matrix(rep(sigma.post, NT - 1), byrow = TRUE, nrow = NT - 1)
          x <- t(x)
          Psi.temp <- outer(sgn, sgn, "*") * Psi
          pr.temp <- pmnorm(x = x, mean = rep(0, NT - 1), varcov = Psi.temp)
          out <- out + pr.temp
        }
      }
      if(r == NT){
        x <-
          (matrix(rep(d.post[k,], NT - 1), byrow = TRUE, nrow = NT - 1) -
             d.post[-k,])/
          matrix(rep(sigma.post, NT - 1), byrow = TRUE, nrow = NT - 1)
        x <- t(x)
        out <- pmnorm(x = x, mean = rep(0, NT - 1), varcov = Psi)
      }
      pr.rank.new.post[k, r,] <- out
      pr.rank.new.w.post[k, r,] <- out * r
    }
  }

  E.rank.new.post <- SUCRA.new.post <-
    matrix(NA, nrow = NT, ncol = iter.post)
  E.rank.new <- SUCRA.new <- matrix(NA, nrow = NT, ncol = 4)
  colnames(E.rank.new) <- colnames(SUCRA.new) <-
    c("Mean", "Median", "95% CrI LB", "95% CrI UB")
  for(k in 1:NT){
    E.rank.new.post[k,] <- colSums(pr.rank.new.w.post[k,,])
    SUCRA.new.post[k,] <- (NT - E.rank.new.post[k,])/(NT - 1)
    E.rank.new[k, 1] <- mean(E.rank.new.post[k,])
    E.rank.new[k, 2:4] <- as.numeric(quantile(E.rank.new.post[k,],
                                          probs = c(0.5, 0.025, 0.975)))
    SUCRA.new[k, 1] <- mean(SUCRA.new.post[k,])
    SUCRA.new[k, 2:4] <- as.numeric(quantile(SUCRA.new.post[k,],
                                          probs = c(0.5, 0.025, 0.975)))
  }

  out <- list(rank = rank, SUCRA = SUCRA,
              rank.post = rank.post, SUCRA.post = SUCRA.post,
              E.rank.new = E.rank.new, SUCRA.new = SUCRA.new,
              E.rank.new.post = E.rank.new.post, SUCRA.new.post = SUCRA.new.post,
              pr.rank.new.post = pr.rank.new.post)
 }else{
  out <- list(rank = rank, SUCRA = SUCRA,
              rank.post = rank.post, SUCRA.post = SUCRA.post)
 }

 if(trace) out$coda <- coda
 return(out)
}
```

Appendix C. Trace plots

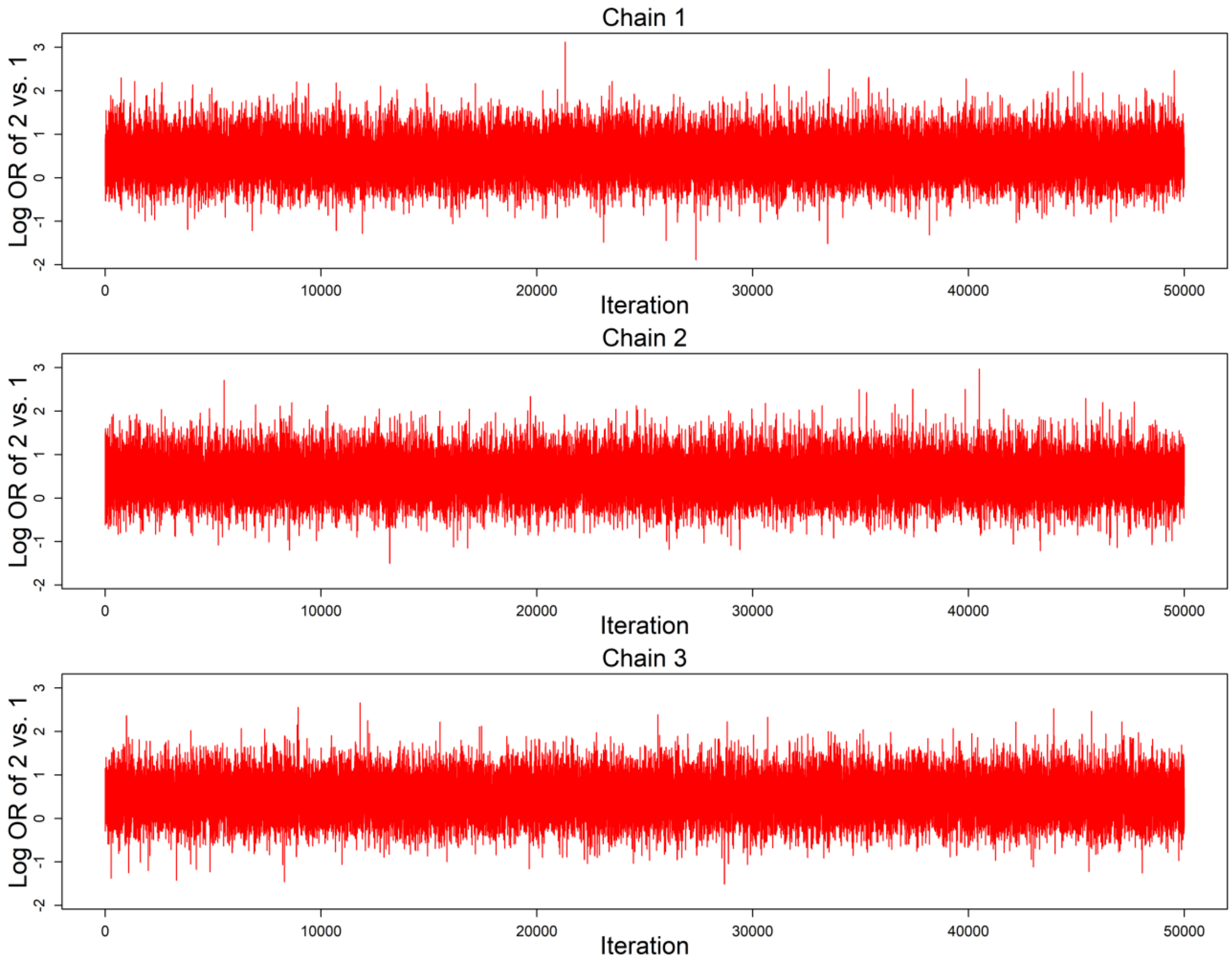

Figure S1. Trace plots of the log odds ratios of treatment 2 vs. the reference treatment 1 in the network meta-analysis on smoking cessation.

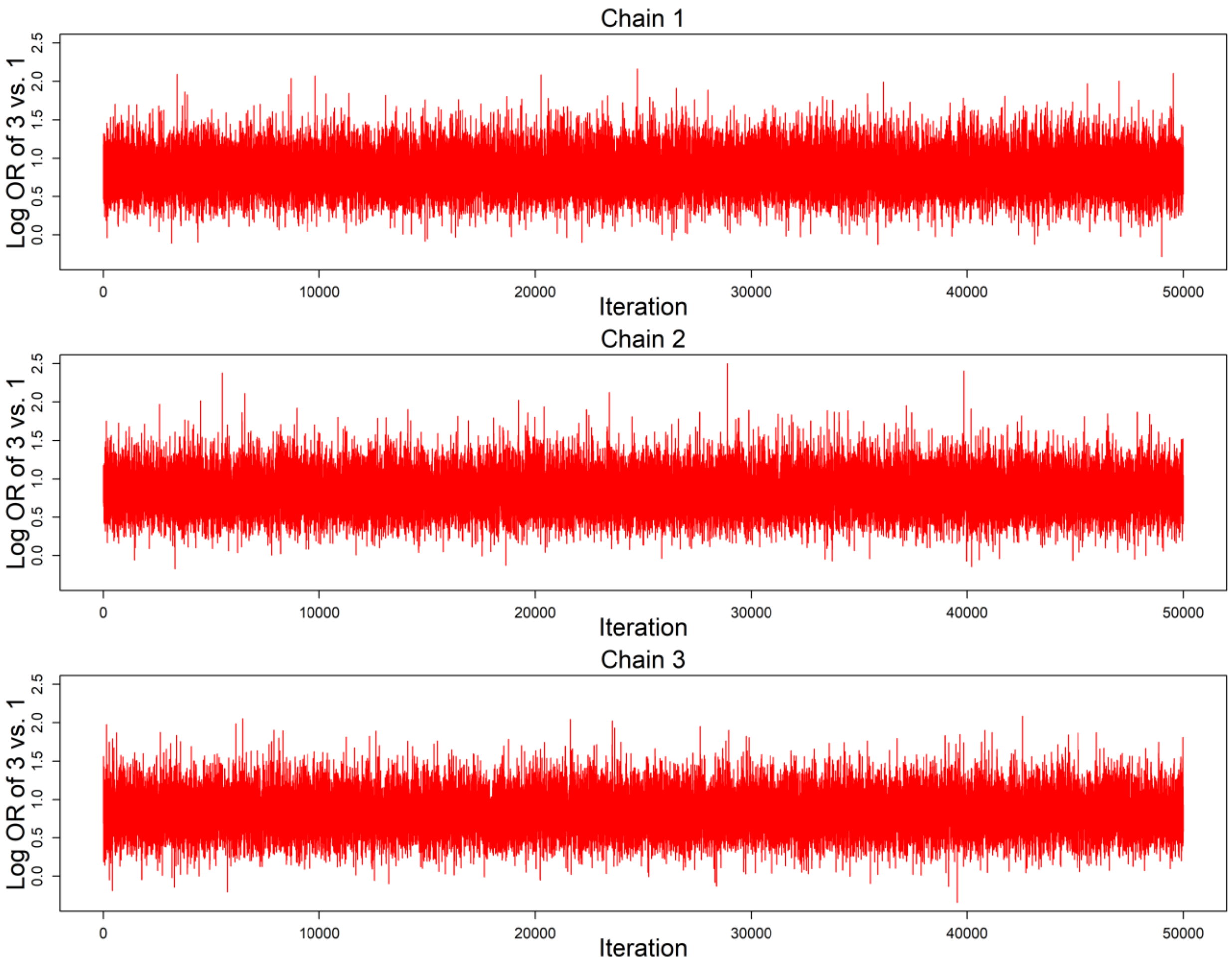

Figure S2. Trace plots of the log odds ratios of treatment 3 vs. the reference treatment 1 in the network meta-analysis on smoking cessation.

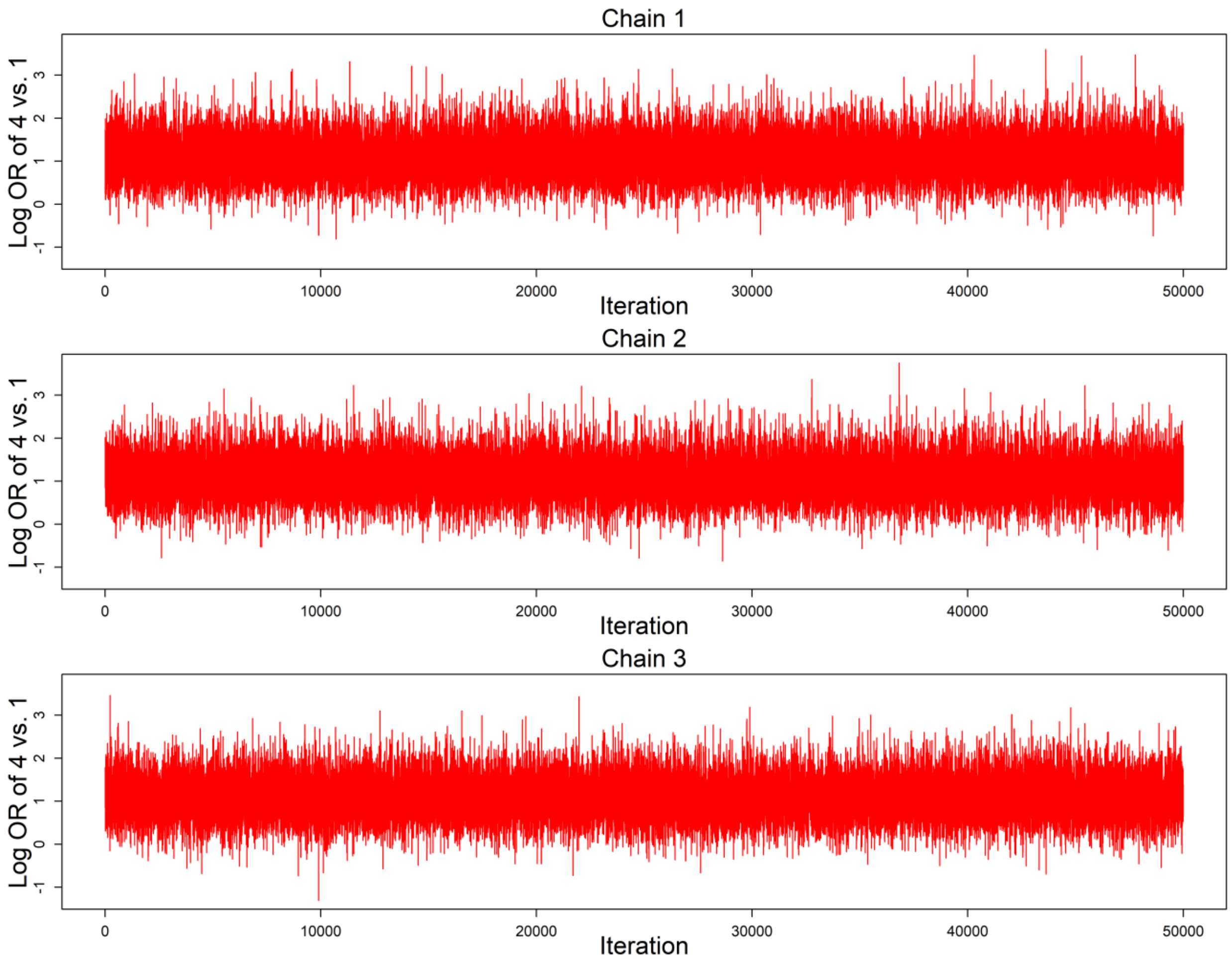

Figure S3. Trace plots of the log odds ratios of treatment 4 vs. the reference treatment 1 in the network meta-analysis on smoking cessation.

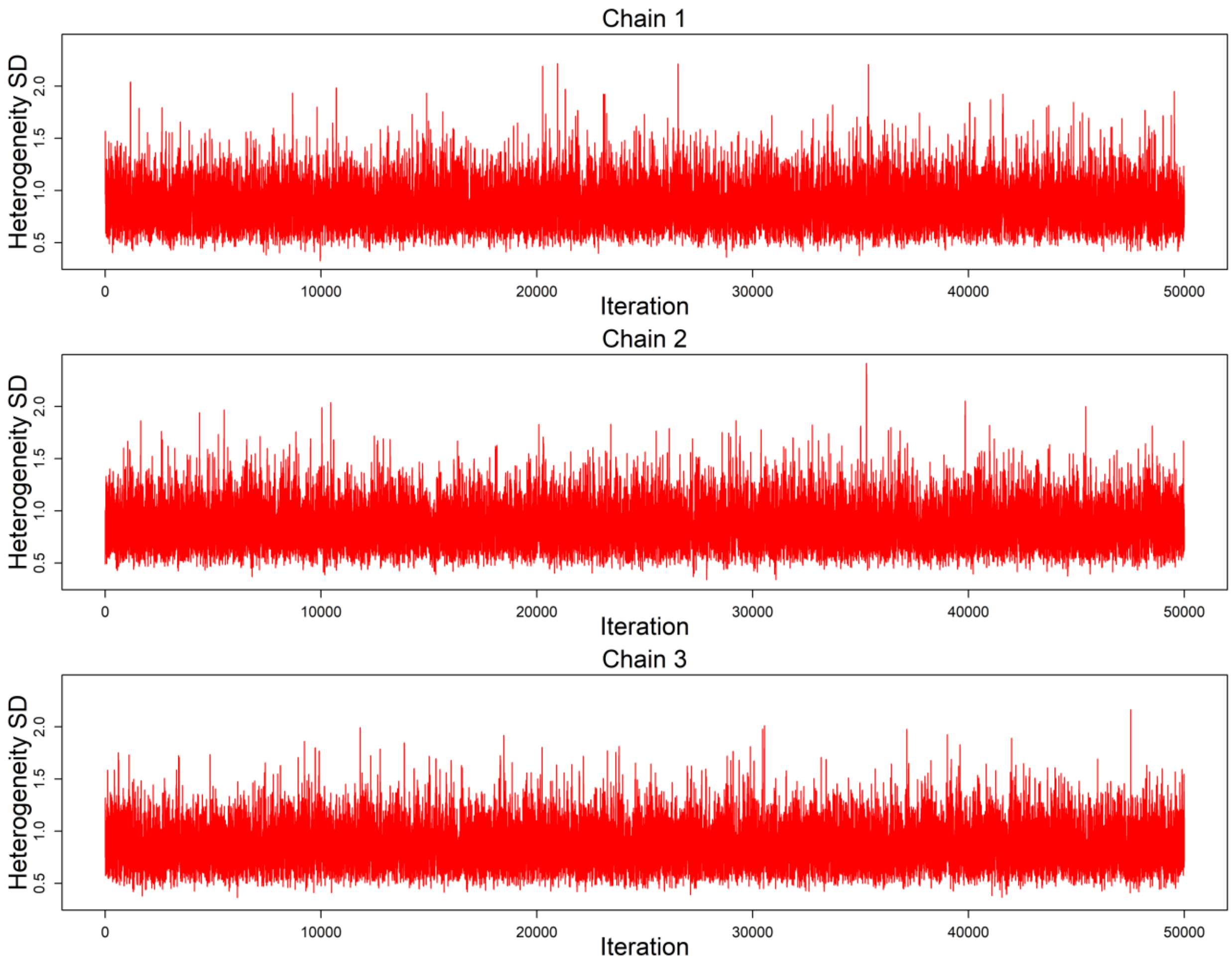

Figure S4. Trace plots of the heterogeneity standard deviation in the network meta-analysis on smoking cessation.

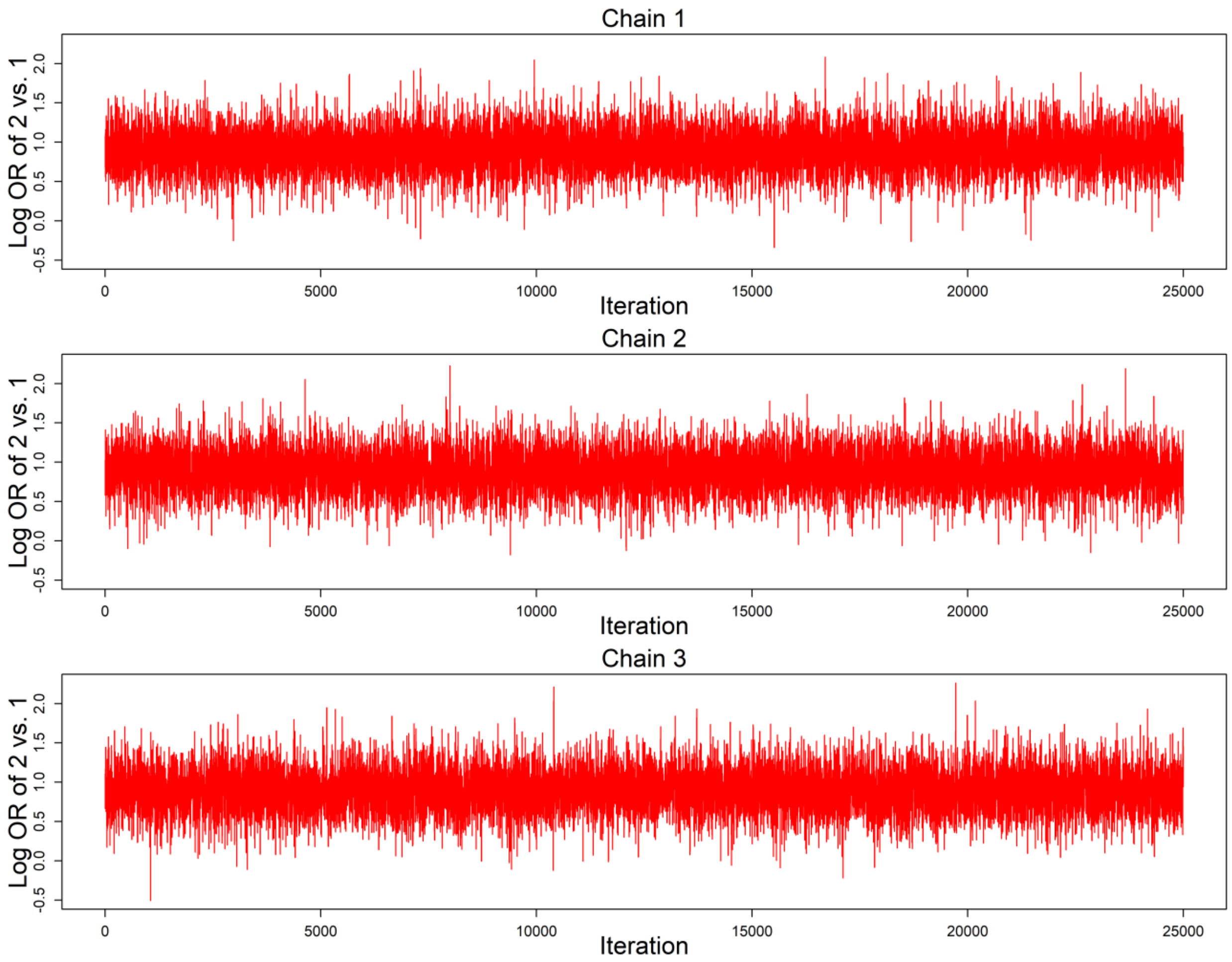

Figure S5. Trace plots of the log odds ratios of treatment 2 vs. the reference treatment 1 in the network meta-analysis on all-grade treatment-related adverse events.

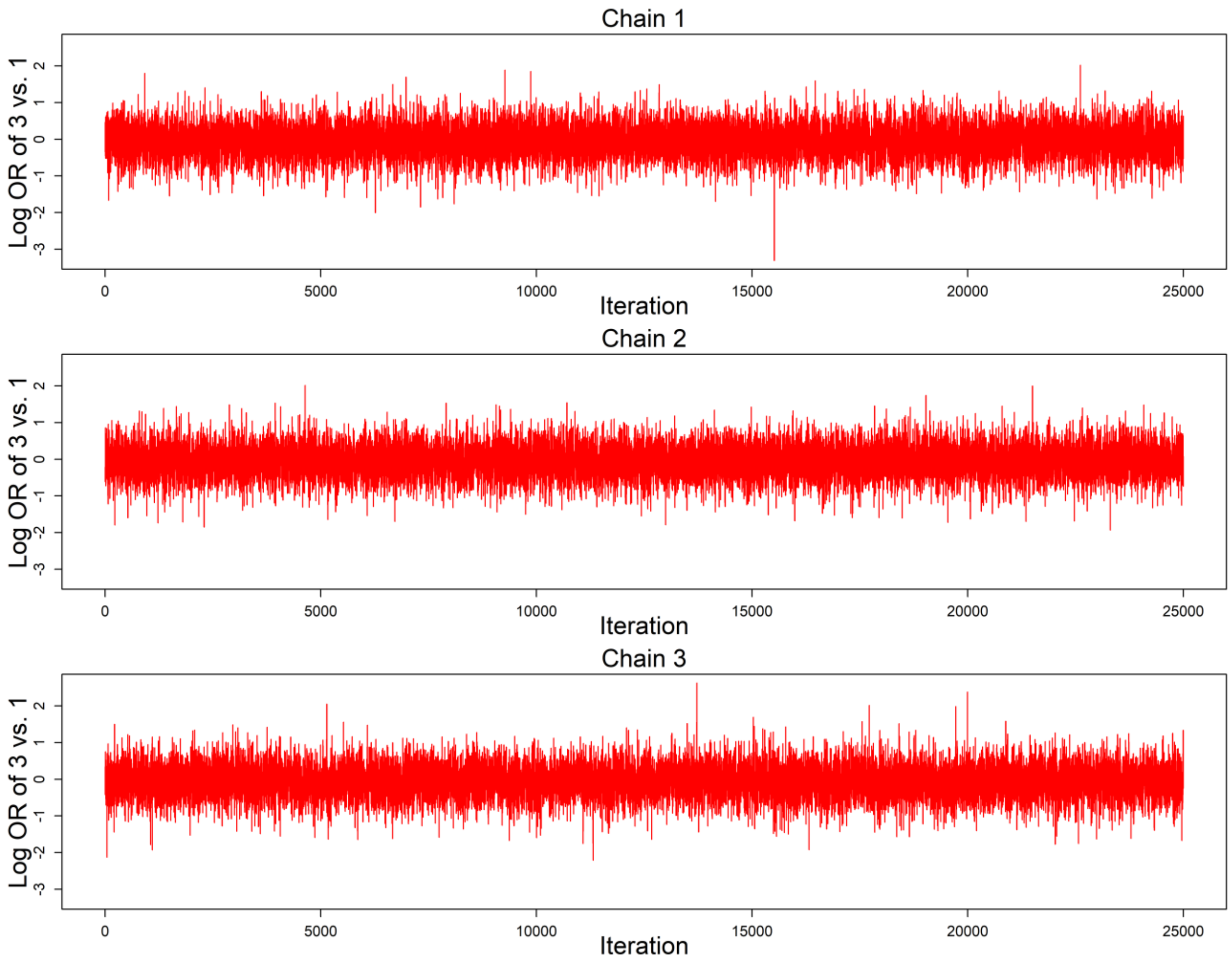

Figure S6. Trace plots of the log odds ratios of treatment 3 vs. the reference treatment 1 in the network meta-analysis on all-grade treatment-related adverse events.

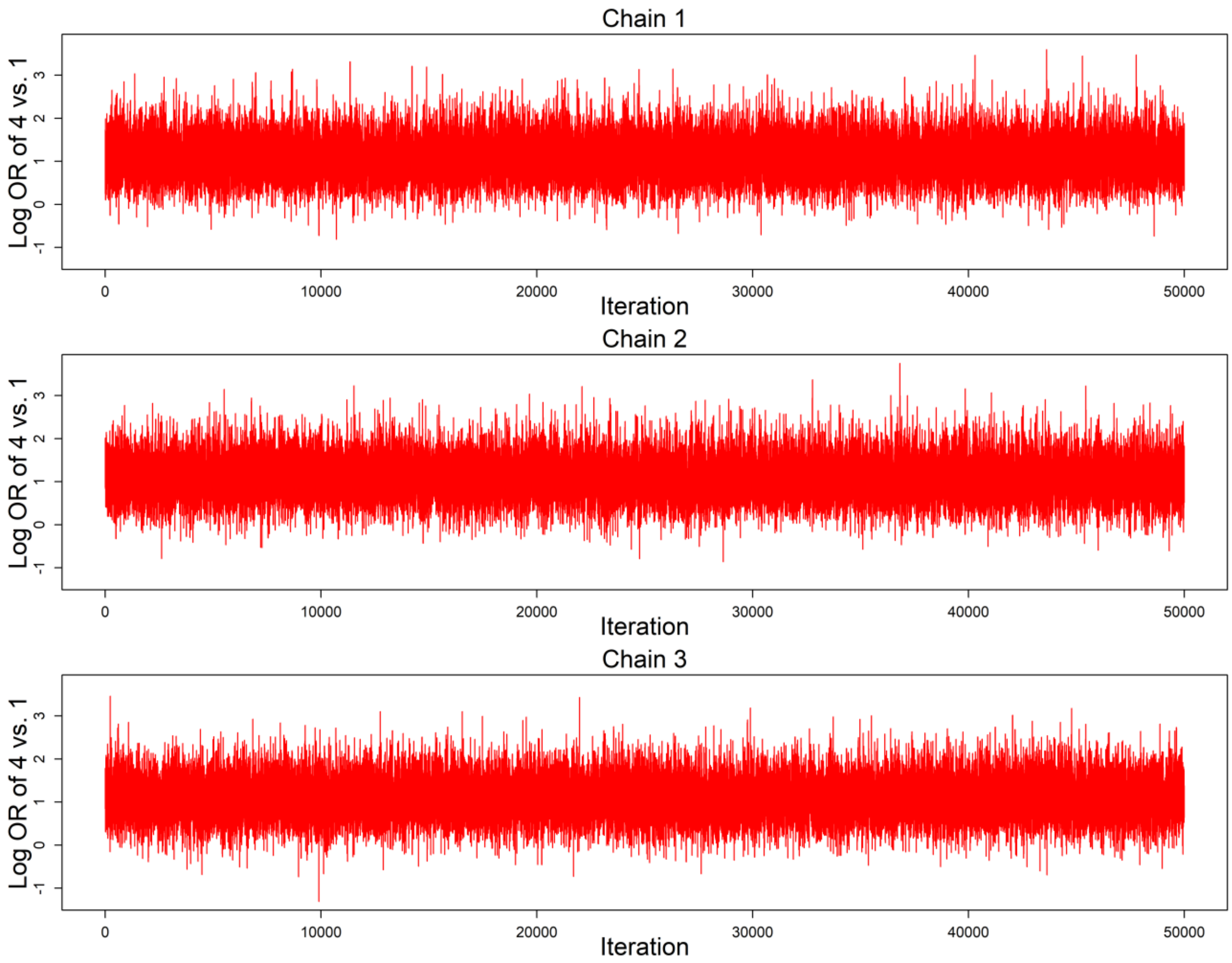

Figure S7. Trace plots of the log odds ratios of treatment 4 vs. the reference treatment 1 in the network meta-analysis on all-grade treatment-related adverse events.

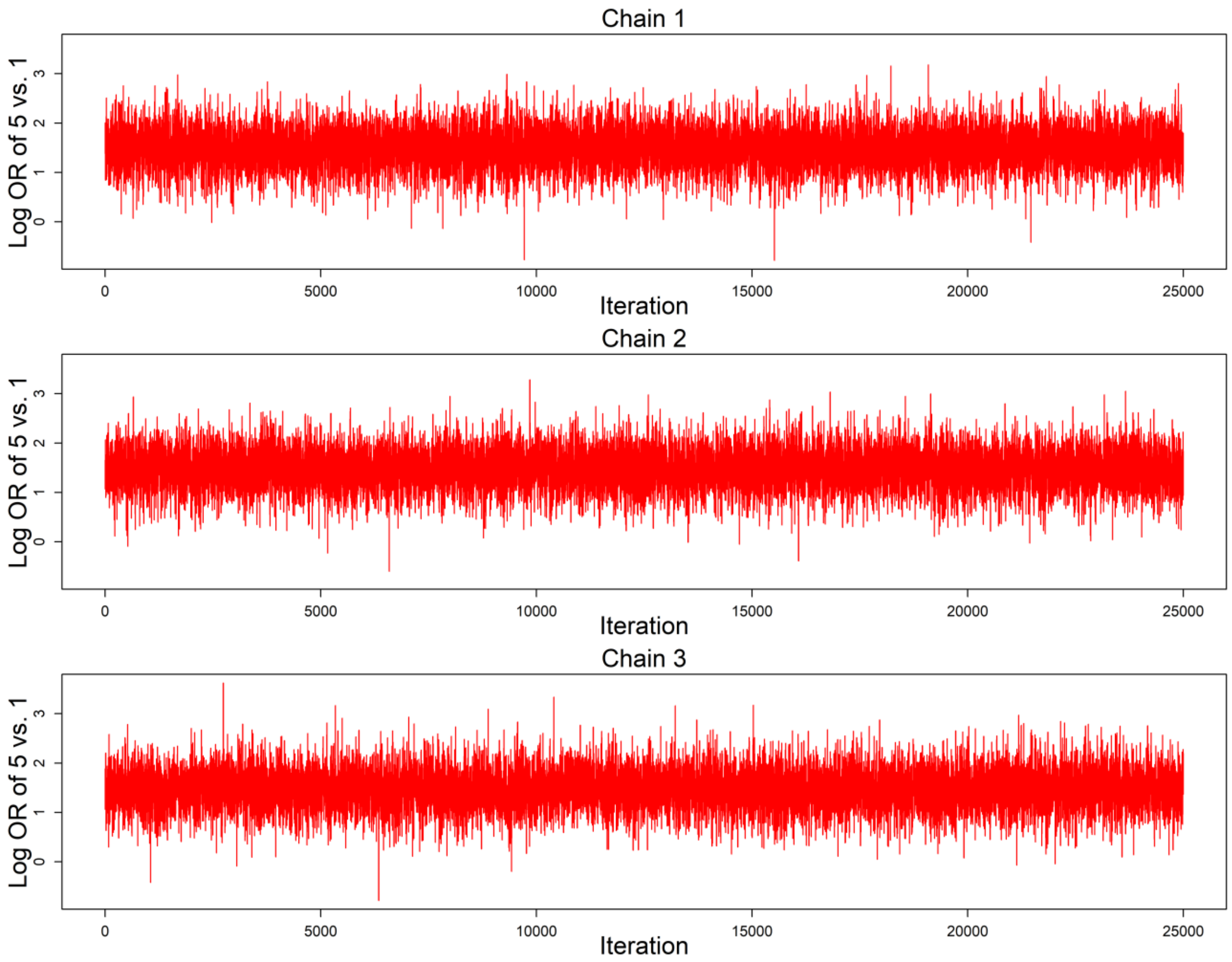

Figure S8. Trace plots of the log odds ratios of treatment 5 vs. the reference treatment 1 in the network meta-analysis on all-grade treatment-related adverse events.

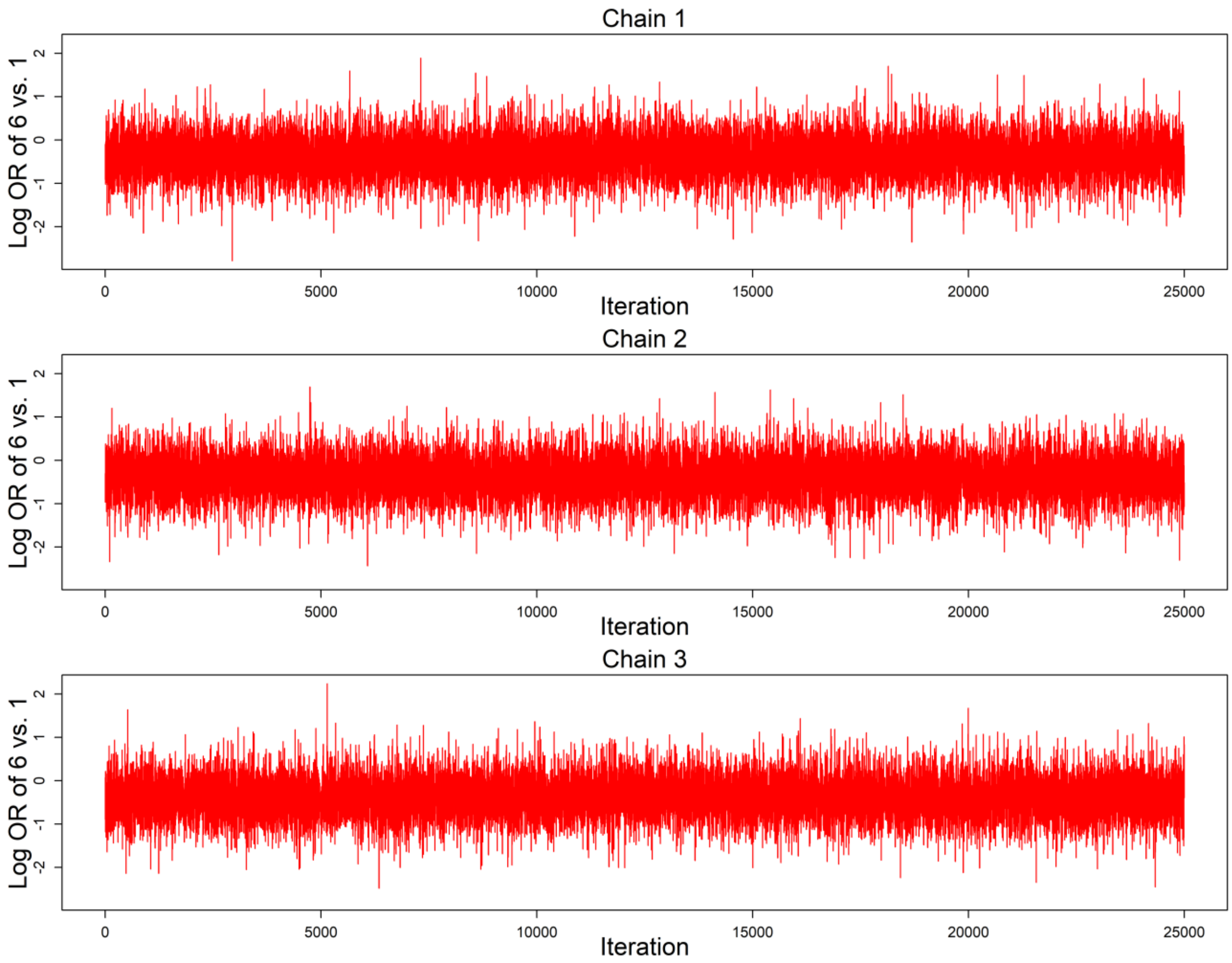

Figure S9. Trace plots of the log odds ratios of treatment 6 vs. the reference treatment 1 in the network meta-analysis on all-grade treatment-related adverse events.

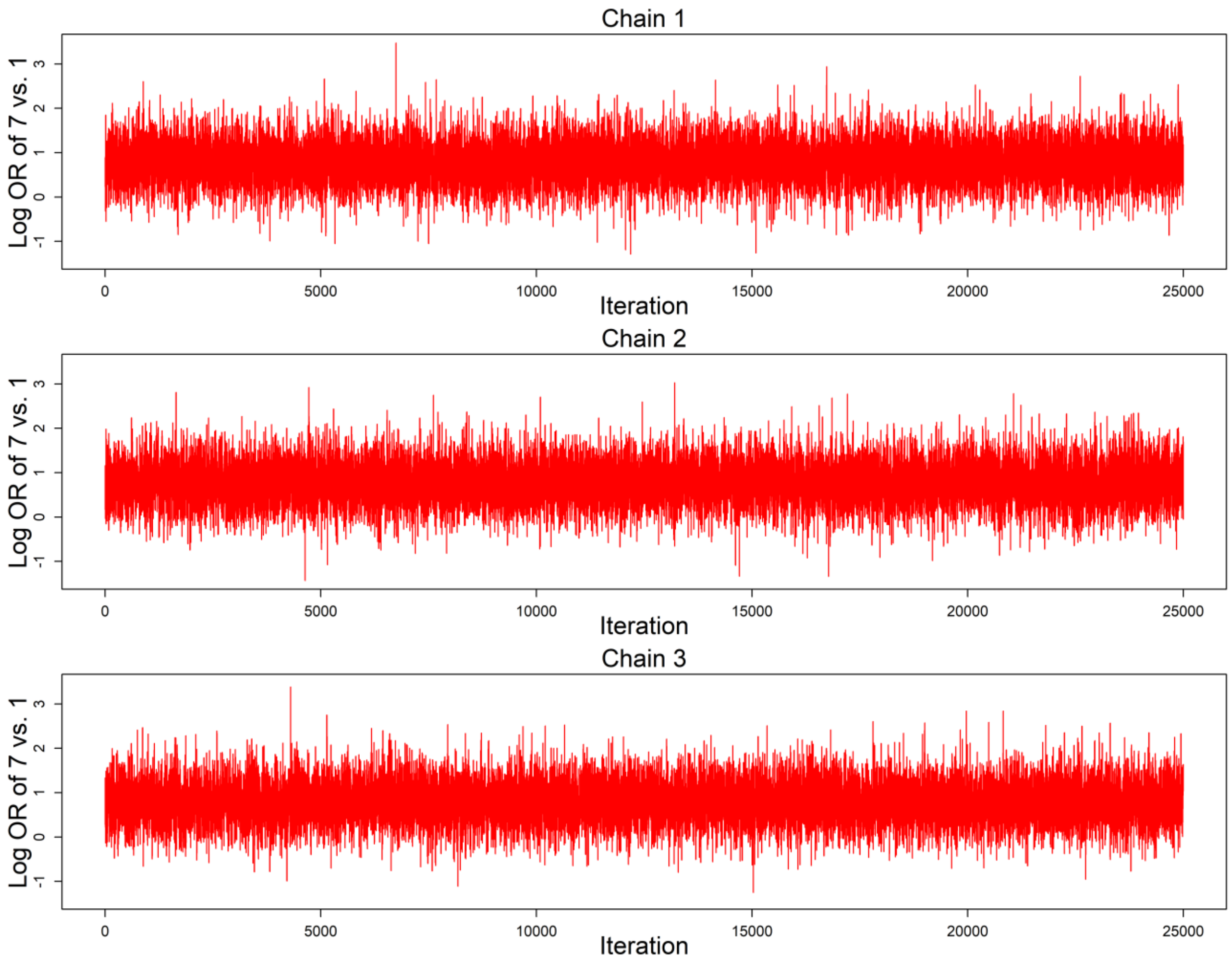

Figure S10. Trace plots of the log odds ratios of treatment 7 vs. the reference treatment 1 in the network meta-analysis on all-grade treatment-related adverse events.

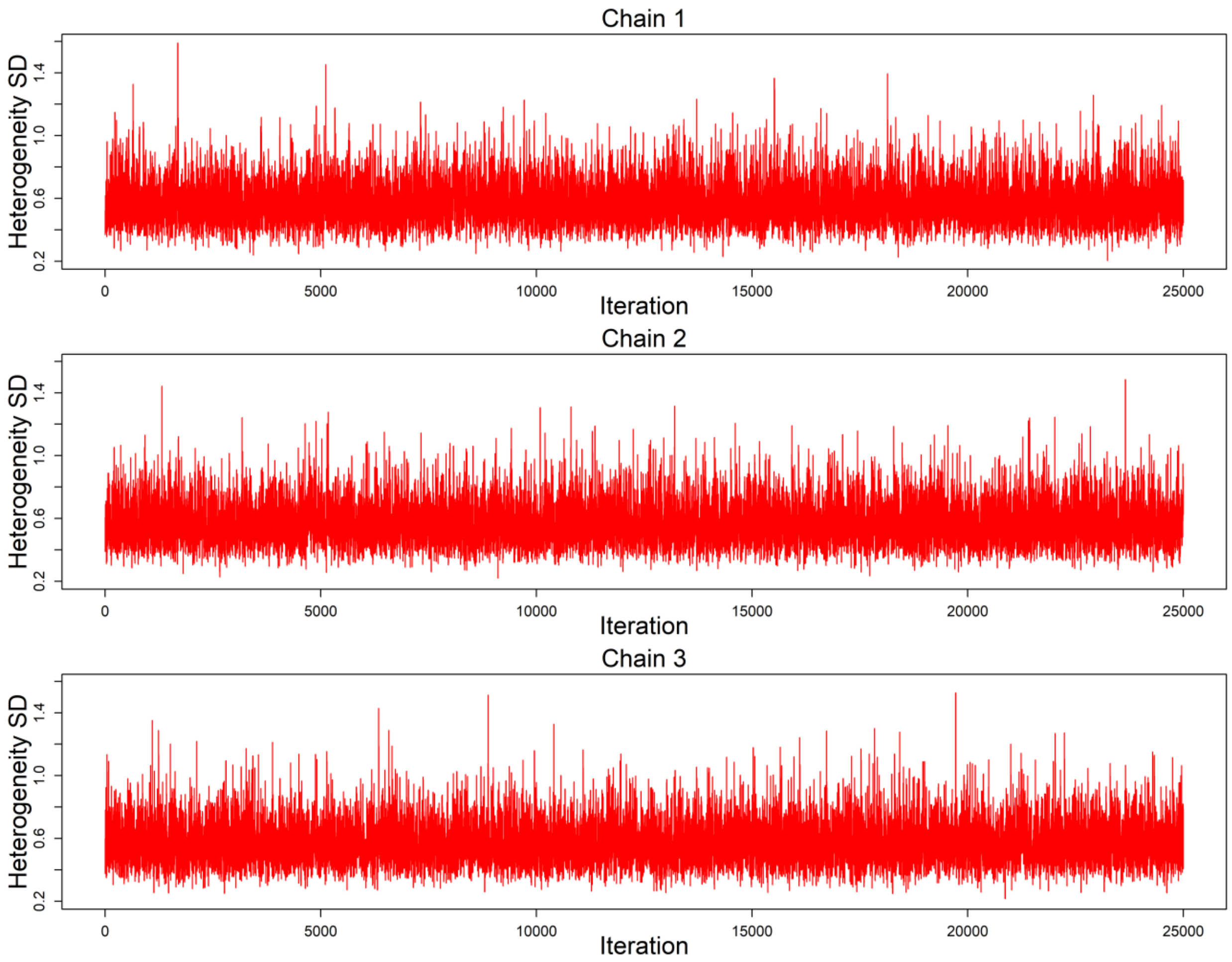

Figure S11. Trace plots of the heterogeneity standard deviation in the network meta-analysis on all-grade treatment-related adverse events.

Appendix D. Secondary analyses

Table S3. Treatment ranking measures produced by the informative prior for the heterogeneity variance in the example of smoking cessation.

Treatment	Mean (P-score)	Median	95% credible interval
Scaled rank in the NMA:			
1	0.037	0.000	(0.000, 0.333)
2	0.397	0.333	(0.000, 1.000)
3	0.689	0.667	(0.333, 1.000)
4	0.877	1.000	(0.333, 1.000)
Expected scaled rank in a new study:			
1	0.194	0.184	(0.062, 0.382)
2	0.439	0.433	(0.188, 0.719)
3	0.624	0.626	(0.426, 0.815)
4	0.743	0.760	(0.448, 0.941)

Note: The posterior means of the scaled ranks in the NMA are the Bayesian P-scores, and those of the expected scaled ranks in a new study are the predictive P-scores.

Table S4. Treatment ranking measures produced by the informative prior for the heterogeneity variance in the example of all-grade treatment-related adverse events.

Treatment	Mean (P-score)	Median	95% credible interval
Scaled rank in the NMA:			
1	0.361	0.333	(0.167, 0.500)
2	0.764	0.667	(0.667, 1.000)
3	0.780	0.833	(0.500, 1.000)
4	0.164	0.167	(0.000, 0.500)
5	0.092	0.000	(0.000, 0.333)
6	0.924	1.000	(0.667, 1.000)
7	0.415	0.500	(0.000, 0.833)
Expected scaled rank in a new study:			
1	0.361	0.365	(0.190, 0.508)
2	0.748	0.749	(0.589, 0.894)
3	0.757	0.770	(0.491, 0.942)
4	0.202	0.172	(0.008, 0.576)
5	0.141	0.124	(0.009, 0.369)
6	0.873	0.897	(0.613, 0.993)
7	0.418	0.413	(0.154, 0.728)

Note: The posterior means of the scaled ranks in the NMA are the Bayesian P-scores, and those of the expected scaled ranks in a new study are the predictive P-scores.
